# Processing closely spaced lesions during Nucleotide Excision Repair triggers mutagenesis in *E*. *coli*

**DOI:** 10.1371/journal.pgen.1006881

**Published:** 2017-07-07

**Authors:** Régine Janel-Bintz, Rita L. Napolitano, Asako Isogawa, Shingo Fujii, Robert P. Fuchs

**Affiliations:** 1 Biotechnologie et Signalisation Cellulaire, Université de Strasbourg, UMR7242, Illkirch, France; 2 Cancer Research Center of Marseille, CNRS, UMR7258 [Genome Instabilty and Carcinogenesis]; Inserm, U1068; Paoli Calmette Institute, Aix-Marseille Univ, Marseille, France; Université Paris Descartes, INSERM U1001, FRANCE

## Abstract

It is generally assumed that most point mutations are fixed when damage containing template DNA undergoes replication, either right at the fork or behind the fork during gap filling. Here we provide genetic evidence for a pathway, dependent on Nucleotide Excision Repair, that induces mutations when processing closely spaced lesions. This pathway, referred to as Nucleotide Excision Repair-induced Mutagenesis (NERiM), exhibits several characteristics distinct from mutations that occur within the course of replication: i) following UV irradiation, NER-induced mutations are fixed much more rapidly (t ½ ≈ 30 min) than replication dependent mutations (t ½ ≈ 80–100 min) ii) NERiM specifically requires DNA Pol IV in addition to Pol V iii) NERiM exhibits a two-hit dose-response curve that suggests processing of closely spaced lesions. A mathematical model let us define the geometry (infer the structure) of the toxic intermediate as being formed when NER incises a lesion that resides in close proximity of another lesion in the complementary strand. This critical NER intermediate requires Pol IV / Pol II for repair, it is either lethal if left unrepaired or mutation-prone when repaired. Finally, NERiM is found to operate in stationary phase cells providing an intriguing possibility for ongoing evolution in the absence of replication.

## Author summary

In this paper, we report the surprising finding that in addition to the well-known properties of Nucleotide Excision Repair (NER) in efficiently repairing a large number of DNA lesions, NER entails a mutagenic sub-pathway. Our data suggest that closely spaced lesions are processed by NER into a toxic DNA intermediate, i.e. a gap containing a lesion, that leads either to mutagenesis during its repair or to cell death in the absence of repair. The paper describes a new pathway for the generation of mutations in stationary phase bacteria or quiescent cells; it also provides an additional role for Pol IV, the most widely distributed specialized DNA polymerase in all forms of life.

## Introduction

Many endogenous and exogenous chemicals as well as radiations covalently damage DNA leading to major perturbations of DNA metabolism such as in replication and transcription. Cells have evolved sophisticated mechanisms to deal with DNA damage, collectively referred to as DNA Repair mechanisms. In principle, DNA Repair pathways refer to processes that effectively remove DNA lesions. Most repair pathways rely on the double-stranded nature of DNA that allows a damaged strand to be corrected by using the information present in the complementary strand. For instance Nucleotide Excision Repair (NER) uses dedicated enzymes that recognize damaged DNA, incise the damaged strand and remove a short oligonucleotide encompassing the damage. Regulation of the NER pathway requires high specificity in lesion recognition by means of molecular matchmaking and kinetic proofreading and proper coordination with the DNA Damage checkpoint response [1–4]. The resulting single-stranded DNA gap is accurately filled-in by a DNA polymerase and sealed by a ligase. This “cut and patch” repair mechanism has a huge effect on protecting cells against genotoxic compounds, indeed an NER-proficient strain is resistant to a UV dose that is almost 50-fold higher compared to an NER-deficient strain [5,6]. Nevertheless, a few lesions may escape repair and will be present at the time of DNA replication, thus perturbing replication fork progression. In that case, cells implement lesion tolerance pathways that are not genuine repair pathways since the lesion is bypassed without being removed [7]. A common lesion tolerance strategy, named Translesion Synthesis (TLS), involves specialized DNA polymerases that are able to copy damaged templates. TLS occurs either directly at the fork or behind the fork during a gap-filling process [8]. Due to the low fidelity of the TLS polymerases, this process is intrinsically error-prone.

In *E*. *coli*, Pol V, encoded by the *umuDC* locus, plays a prominent role in Translesion Synthesis (TLS) and mutagenesis [9,10]. Indeed, essentially all UV-induced mutagenesis is abolished when *umuDC* is inactivated. The primary role of Pol V in UV-induced mutagenesis is due to its unique property to mediate the specific insertion steps across the major UV lesions [11,12].

The early-time responses of *E*. *coli* cells to UV irradiation have been monitored by the kinetics of dNTP incorporation into newly synthesized DNA [13–16]. A common conclusion from these investigations is that, after irradiation of a wild type strain, following an initial abrupt drop, the rate of synthesis increases to progressively reach its normal speed over a period 40–50’ post irradiation. Compared to a wild type strain, inactivation of the TLS polymerases only modestly affects the recovery process [13,14]. This observation can be interpreted as follows: when the replication fork encounters a lesion the fork does not stop permanently. Indeed, when a DNA polymerase encounters a replication-blocking lesion in the leading strand, the replicative helicase continues unwinding the two strands but at a highly reduced rate due to its uncoupling from the DNA polymerase [17,18] before downstream re-priming eventually occurs [19]. Downstream re-priming events create gaps that are subsequently repaired most often by recombination with the sister chromatid or more rarely by TLS [20]. The observed reduction in the rate of DNA synthesis may thus reflect the reduced fork speed due to the uncoupling / re-priming process [5,6,21] rather than indicating a complete stop in replication fork progression as often suggested [7,22]. During the ≈ 50’ period, NER removes most of the lesion genome-wide to permit recovery of full synthesis rate [14–16,23]. Not surprisingly, in a *uvrA* strain the rate of synthesis post UV irradiation is strongly affected [14].

The notion that mutagenesis may be associated, at least in part, with NER has been suggested more than 40 years ago by Nishioka and Doudney who reported that UV mutagenesis coincides with the loss of photoreversibility of UV lesions within a 20 min post UV irradiation period in a wild-type but not in a *uvrA* strain [24,25]. These data were interpreted as evidence for a NER-dependent pathway that induces mutations in a wild-type strain at early time points following UV irradiation. Bridges extended this observation by showing that the NER-dependent mutation pathway was recA+ dependent [26]. Moreover, evidence for NER-dependent UV mutagenesis was described *in vitro* in *E*. *coli* crude extracts [27,28]. In NER-proficient *S*. *cerevisiae*, it was shown that mutations can occur prior to the first post-UV replication cycle [29] [30] while mutations are not fixed prior the first post-UV irradiation replication cycle in NER-deficient yeast cells, [31]. Studies on the repair of clustered lesions in double-stranded DNA have mostly been restricted to lesions induced by radiations such as Uracil residues, abasic sites or 8-oxo-G which are typical substrates for Base Excision Repair pathways (for a review see [32]). The concept that closely spaced UV lesions are a potential source of mutations has been documented and discussed in bacteria [33,34] and in yeast where it was shown that mutations accumulate as a function of the square UV dose in wild-type cells but not in NER-deficient cells [30]. More recently, the occurrence of UV-induced mutations in non-proliferating yeast cells prior to replication was demonstrated [35]. While this class of mutations is characterized by the occurrence of the mutation in both DNA strands prior to replication in a NER-dependent way, the data did not allow distinguish between a linear or a quadratic dose-response curve. Despite all these data, the precise mechanistic and genetic links between NER and mutagenesis have largely remained unexplored.

In the present paper, we show that in NER-proficient strains, a significant fraction of UV-induced mutagenesis occurs within the time frame of active NER, i.e. when replication is shutdown. Interestingly, these early time-point mutations disappear in NER-deficient strains. Thus, in proliferating cells, UV-induced mutations fall in two classes, mutations induced during replication (RiM) and NER-induced mutagenesis (NERiM), respectively.

We also show that mutations induced in a NER-dependent way accumulate with the square of the UV dose suggesting a “two-hit mechanism”. Normally, the NER machinery excises lesions as small damage-containing oligonucleotides and leaves a short gap that is filled-in; however, if following an initial NER incision event, another lesion resides in close proximity in the complementary strand, the second lesion may become exposed within the initial NER gap forming a so-called opposing lesion structure. The second lesion may be converted into a point mutation during filling-in of the NER associated gap. The mutagenic process associated with NER not only requires Pol V, but also DNA Pol IV and to some extent of Pol II. Additionally, we would like to stress that when two lesions are located in close proximity in the same strand, processing of one of them by NER may also trigger gap enlargement and subsequent requirement for processing by Pol IV/Pol II. For sake of simplicity, we will essentially discuss the situation were the closely spaced lesions are located in opposite strands.

Our data are consistent with NERiM playing a significant role in UV mutagenesis in proliferating cells; indeed, even at relatively low levels of irradiation (40J/m^2^), NERiM accounts for 1/3 of UV-induced mutagenesis. In addition we show that the role of NERiM becomes predominant in stationary phase bacteria suggesting an elegant way to generate genetic diversity in non-replicating microbes and perhaps in post-mitotic metazoan cells.

## Results and discussion

### Genetic description of a NER-dependent mutagenesis pathway (Fig 1)

**Fig 1:**
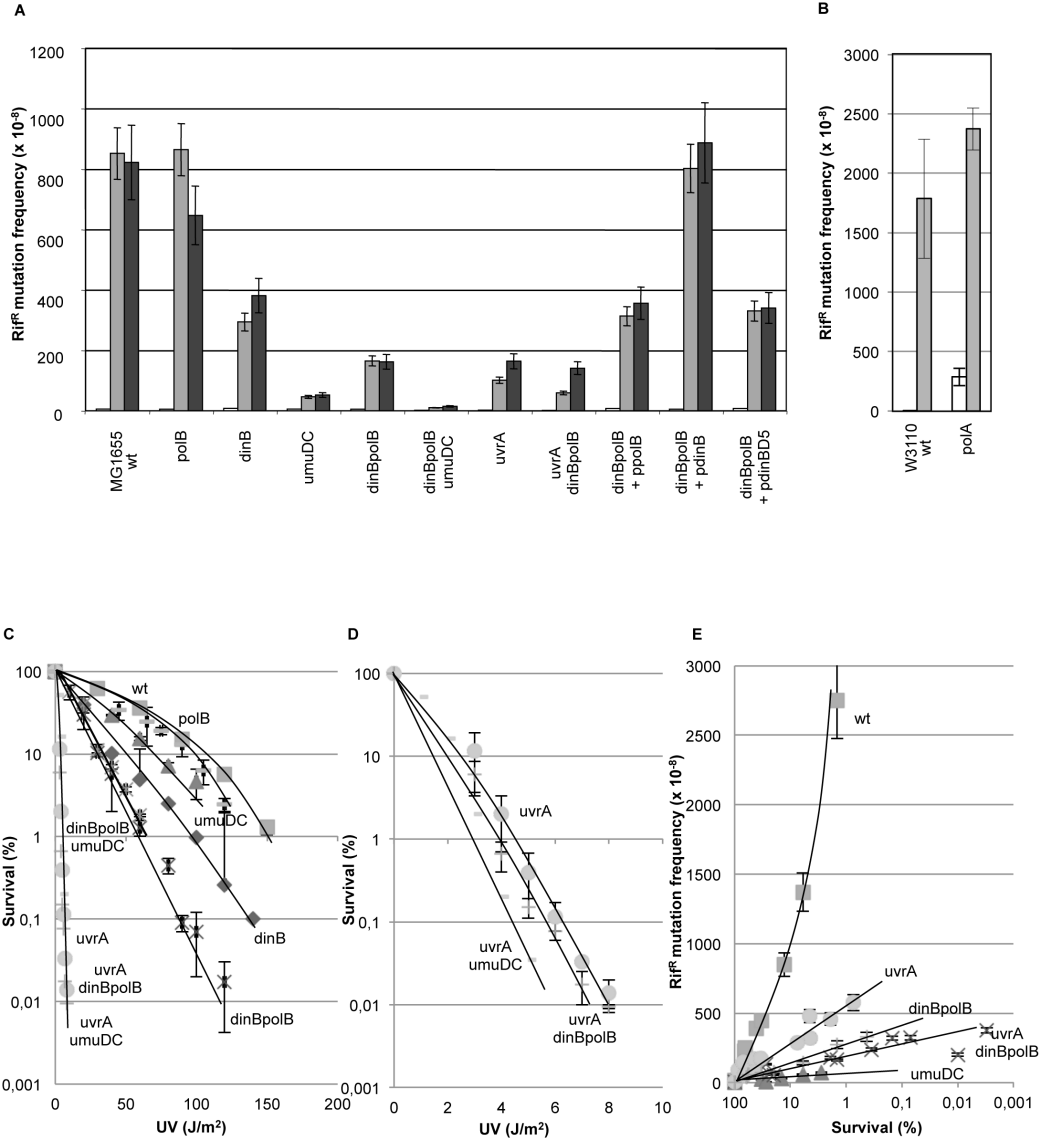
Genetic interactions define a mutation pathway that is dependent upon *dinBpolB* and nucleotide excision repair genes. **1A**: Rif^R^ mutation frequencies were determined in various strains in response to UV irradiation. All strains in fig 1A are constructed in the MG1655 background. To account for the intrinsic differences in UV sensitivity among strains, we compared UV doses leading to similar levels of survival: grey bars correspond to UV doses leading to survival levels ranging between 5–15%, for black bars survival levels range between 1–5% survival. It should be stressed that at these UV doses, the SOS response is fully induced in all strains. White bars represent the level of spontaneous mutation frequency, i.e. no UV irradiation. Average values and standard deviations are plotted for three or more independent experiments per strain. **1B**: The *polA1* allele data are presented in a separate panel as the background in which this allele resides is w3110. Background w3110 exhibits a ≈2-fold higher UV-induced mutagenic response compared to the MG1655 background at UV irradiation levels leading to similar survival. Grey bars correspond to UV doses leading to survival levels ranging between 5–15%; white bars represent the level of spontaneous mutation frequency, i.e. no UV irradiation. **1C**: Survival curves of DNA polymerase mutant strains. Wild-type: ■squares; *polB*: **−** (horizontal segment); *umuDC*: Δ triangles; *dinB*:◆ losanges; *dinBpolB*: X crosses; *dinBpolBumuDC*: ★ stars. **1D**: Survival curves of DNA polymerase mutant strains in an NER-defective background: *uvrA* •(dots), *uvrAdinBpolB* ✚ (crosses), *uvrAumuDC*: **−** (horizontal segment). **1E**: UV-induced mutation frequencies plotted as a function of survival in various strains. Wild-type strain ■(squares), *uvrA* •(dots), *dinBpolB* X (crosses), *uvrAdinBpolB* ✚(crosses), *umuDC* Δ (triangles).

#### A *dinBpolB*-mediated mutagenesis sub-pathway

We wanted to revisit the involvement of SOS-controlled DNA polymerases in UV-induced mutagenesis in *E*. *coli*. For that purpose we compared UV-induced mutagenesis in various polymerase defective strains (Table 1) using the rifampicin resistance mutation assay [9,10,36]. This assay is able to monitor all 6 types of base substitutions at 27 different sites within a short fragment, between amino acids 500 to 580, of the *rpoB* gene product [37]. Over 90% of rif^R^ mutants were shown to map within this 250 bp sequence [36]. Given that DNA polymerase mutant strains are distinctly more UV-sensitive than wild-type *E*. *coli*, we choose to compare the different strains at UV-doses leading to similar levels of survival. For each strain, we choose two UV doses leading to 5–10% and 1–5% survival ranges, respectively (Fig 1A). Typically, wild-type *E*. *coli* cells exhibit a robust increase in the frequency of rif^R^ mutant colonies upon irradiation with UV light (Fig 1A) from ≈ 2–8 x 10^−8^ in the absence of irradiation (spontaneous mutation frequency) to ≈ 800–1000 x 10^−8^ at a UV dose that reduces survival to ≈ 5–10% (Fig 1E). Pol V, the specialized DNA polymerase encoded by the *umuDC* operon is the major bypass polymerase in *E*. *coli* [9,11,12,38,39]. Indeed, there is little residual mutagenesis in a *umuDC* strain compared to a wild-type strain at all UV doses (Fig 1A and 1E). Surprisingly however, a severe reduction in mutagenesis (≈ 4 fold) is observed in a double *dinBpolB* mutant strain, defective in DNA polymerases IV and II (Fig 1A) suggesting the existence of a *dinBpolB* sub-pathway for induced mutagenesis. Pol IV appears to play a dominant role in this sub-pathway since a two-fold reduction in induced mutagenesis is observed in the single *dinB* mutant strain, while no effect is observed in a single *polB* strain. Introduction of low copy number plasmids expressing Pol II or Pol IV into the *dinBpolB* strain restores mutagenesis to the levels observed in *dinB* or *polB* strains respectively (Fig 1A). Taken together the data suggest that Pol V controls essentially all UV-induced mutagenesis; however, a sub-fraction of UV-mutagenesis, additionally, requires Pol IV (and Pol II) in a yet undefined role. While Pol II has no effect on its own, it partially compensates for the lack of Pol IV. For the sake of being complete we also checked UV mutagenesis in a strain defective in Pol I activity (*polA1*); no reduction in induced mutagenesis compared to the corresponding wild-type strain was observed (Fig 1B). However, as previously noted, the *polA1* strain exhibits a higher level of spontaneous mutagenesis [14–16,23,40].

**Table 1:** Strains and plasmids.

**Strain**	
MG1655	rph1 rfb50 ilvG1
MG1655 Δ*polB*:: Ω	as MG1655, ΔpolB strepR/specR (Y. Matic)
MG1655 Δ*dinB*:: Kan	MG1655 x P1. YG7210 (this work)
MG1655 Δ*umuDC*:: Cm	MG1655 x P1. YG7210 (this work)
MG1655 Δ*dinB*::Kan Δ*polB*::Ω	MG1655 ΔpolB x P1. YG7210 (this work)
MG1655 Δ*dinB*::Kan Δ*polB*::Ω Δ*umuDC*::Cm	MG1655ΔpolBΔdinBxP1.YG7210(cl.2),MG1655ΔpolBΔumuDCxP1.YG7210 (cl.3) (this work)
MG1655 *uvrA*:: Tn10	MG1655 x BH200 uvrA::Tn10 (this work)
MG1655 *uvrA*:: Tn10 Δ*dinB*::Kan Δ*polB*:: Ω	MG1655 ΔdinB ΔpolB x BH200 uvrA::Tn10 (this work)
MG1655 Δ*polB*:: Kan	MG1655 x P1. YG2241 ΔpolB::Kan (this work)
MG1655 Δ*dinB61*:: ble *	MG1655 x P1. AR30 ΔdinB61::Ble (this work)
MG1655 Δ*polB*:: Kan Δ*dinB61*:: ble *	MG1655 ΔdinB61::Ble x P1. YG2241 ΔpolB::Kan (this work)
MG1655 *dnaB8ts mal*::Cm	MG1655 x JJC809 *dnaB8ts mal*::Cm
MG1655 Δcho::Cm(Frt)	MG1655 x P1. CS5540
MG1655 Δcho(CmS)	MG1655 Δcho::Cm(Frt)/pCP20 -> Δcho(CmS)
CS5540	KMBL1001 Δcho::Cm(Frt) (N. Goosen)
MG1655 ΔuvrC::Cm	MG1655 x P1.MG1655 ΔpolBΔdinB uvrC::Cm
MG1655 ΔpolB ΔdinB ΔuvrC::Cm	MG1655 ΔpolB ΔdinB x P1. CS5430
CS5430	KMBL1001 ΔuvrC::Cm
MG1655 Δcho(CmS) ΔuvrC::Cm	MG1655 Δcho(CmS) x P1. MG1655 ΔpolB ΔdinB uvrC::Cm (N. Goosen)
MG1655 ΔpolB ΔdinB Δcho(CmS) ΔuvrC::Cm	MG1655 ΔpolB ΔdinB Δcho(CmS) x P1. MG1655 ΔpolB ΔdinB uvrC::Cm
W3110 wt (from *CGSC*)	F-, *λ*^*-*^, *thyA36*, *IN(rrnD-rrnE)1*, *rph-1*, *deoC2*
P3478 *polA1* (CGSC)	F-, *λ*^*-*^, *thyA36*, *IN(rrnD-rrnE)1*, *rph-1*, *deoC2*, *polA1*(Am)
YG7210	AB1157 ΔdinB ΔumuDC (T. Nohmi)
BH200	AB1157 uvrA::Tn10 (S. Boiteux)
YG2241	AB1157 x P1.HRS6700 (T. Nohmi)
AR30	thr1D(gpt-proA)62 hisG4 argE3 araD139 lacY1 galK2 xyl5 mtl1 dinB61::ble sulA211 supE44 tsx33 rpsL31 thi1 (R. Woodgate)
**plasmids**	
pYG768 *dinB* (Ap) pWSK29	(T. Nohmi)
pYG787 *polB* (Ap) pWKS30	(T. Nohmi)
pGB2 o^c^-*dinB*	(this work)
pGB2 o^c^-*dinB*Δ5	(this work)

By which mechanism may Pol IV/Pol II participate in UV-induced mutagenesis? Although most UV-mutagenesis requires functional Pol V (Fig 1A and 1E) the possibility remains that a subset of UV-induced lesions may require Pol IV and/or Pol II, in addition to Pol V, for TLS. Concerning the known major UV-induced lesions, namely the cis-syn pyrimidine cyclobutane dimer (CPD) and the (6–4) pyrimidine-pyrimidone photoproduct, it has been shown that Pol V is both necessary and sufficient for the mere TLS step [11,12,17,18]. We reasoned that if bypass of a particular subset of UV-induced lesions is mediated by Pol IV and Pol II, these lesions are likely to exhibit a distinct mutagenic signature. To investigate this possibility we compared the rif^R^-induced mutation spectra in wild-type and *dinBpolB* strains. In both wild-type and *dinBpolB* strains, the spectra show that most mutations (≈90%) are produced at di-pyrimidine sites with the expected mutagenic signature of Pol V (i.e. 60–70% of transitions, mostly C->T, [11] (S1A and S1B Fig). In conclusion, our data suggest that the role of Pol IV/Pol II in UV-induced mutagenesis does most likely not involve Pol IV and/or Pol II at the TLS step *per se*, at the exception of mutations at codon 526 that will be discussed below.

#### A *dinBpolB*-mediated survival pathway

Next we considered the possibility of Pol IV/Pol II being involved in a gap-filling step. This prompted us to analyze UV-survival curves in strains with defects in these TLS polymerase genes (Fig 1C and 1D). Compared to a wild type strain, an isogenic *polB* strain is not significantly UV sensitive. On the other hand, *umuDC* and *dinB* mutant strains are slightly more UV sensitive. A similarly modest UV sensitivity has previously been reported [14] for the same *dinB* strain (allele: ΔdinB::kan, [41]). This *dinB* allele is further sensitized upon introduction of a *polB* mutation (Fig 1C). It should be noted that a novel *dinB* allele, Δ*dinB883*, that derives from ΔdinB::kan by a spontaneous +1 mutation in a run of 11 G’s was recently described [42]. It exhibits a gain-of-function phenotype that results in a dramatic 100 fold increased UV sensitivity [42]. Given the UV survival data (Fig 1C), the *dinB* allele used in the present work corresponds to the original ΔdinB::kan allele ([41]). The triple *umuDCdinBpolB* mutant strain is equally UV-sensitive as the *dinBpolB* strain. Interestingly, the *dinBpolB* mediated UV-sensitivity observed in the wild-type background (i.e. NER+ background) is no longer seen in an NER-defective (*uvrA*) background, as *uvrA* and *uvrAdinBpolB* survival curves are superimposed (Fig 1D). A *uvrAumuDC* strain is slightly more sensitive than *uvrA* illustrating the contribution to survival of Pol V-mediated TLS during replication (Fig 1D). Taken together these results suggest the existence of a *dinBpolB*-mediated survival pathway that is only effective when NER is functional; in other words, the biological role of the *dinBpolB*-mediated survival pathway is to tolerate DNA structures generated during NER (the *dinBpolB* pathway is epistatic to NER for UV survival). The nature of the DNA structure that requires processing by the *dinBpolB* pathway will be discussed below.

#### The *dinBpolB*-mediated mutagenesis sub-pathway is epistatic to NER

To further explore the relationship between NER and *dinBpolB*, we conducted UV-induced mutagenesis experiments in NER-deficient strains. At first, we monitored the mutation frequencies in various strains irradiated at two UV doses leading to similar levels of survival (Fig 1A: 5–10% and 1–5% survival ranges, respectively). However, due to severe differences in UV sensitivity among NER-deficient and NER-proficient strains, we established full dose-response curves by plotting induced mutation frequencies as a function of UV survival rather than as a function of UV dose (Fig 1E). The rationale for this choice is discussed in the Conclusion paragraph. Remarkably, at any given level of survival, the extent of UV-induced mutations is much higher in a wild-type strain than in the corresponding *dinBpolB* strain confirming the existence of a UV-induced mutagenesis pathway that depends upon Pol II/Pol IV (Fig 1E). The dose-response curves in *dinBpolB* and in *uvrApolBdinD* strains are essentially superimposed, suggesting that the *dinBpolB* pathway fully depends upon NER. The major NER associated endonuclease *uvrC*, but not its homolog *cho* [43], also displayed epistasis to *dinBpolB* with respect to UV-induced mutagenesis (S2 Fig). We also note that both *dinBpolB* and *uvrAdinBpolB* curves are located below the *uvrA* dose-response curve suggesting the existence of a minor mutagenesis pathway that is *dinBpolB*-dependent but NER-independent. This pathway may involve a particular class of UV lesion that specifically requires Pol IV/Pol II for TLS during replication. In support to this hypothesis, there is a specific rif^R^ mutation site (GC->TA at codon position 526), not involving a PyPy sequence, which appears to be a mutation hot spot in the wild-type (4 independent occurences) but not in the *dinBpolB* strain (no occurrence) (S1A and S1B Fig). In order to assess the statistical significance of this observation we applied the Chi-square homogeneity test [44] to the wild type and *dinBpolB* mutation distributions. The calculated chi-square value (with 7 degree of freedom) is equal to 14.2 leading to a p-value of ≈0.05 that suggests the two spectra are likely to be distinct. However, the same statistical analysis suggests the wild-type and *dinBpolB* spectra to be similar provided position 526 is excluded. Indeed, when applying the Chi-square homogeneity test to wild type (excluding mutations at position 526) and *dinBpolB* mutation spectra, the chi-square value is equal to 10.2 (with 6 degree of freedom) leading to a p-value of 0.1 suggesting that there is no reason to consider the two spectra to be different. We hypothesize that this particular sequence context may host a particular class of UV lesions that remains to be identified and that specifically requires Pol II / Pol IV for its bypass.

#### Defining NER-dependent and NER-independent mutagenesis pathways

The difference in the level of UV-induced mutagenesis between the *uvrA* and wild-type strains (Fig 1E) suggests the existence of two UV mutagenesis pathways: one that is NER-dependent (NERiM) and another that is NER-independent, to which we will refer to as the replication-induced (RiM) pathways. In a NER-proficient (wild-type) background, the observed mutation frequency is the sum RiM+NERiM, while the mutations observed in a NER-deficient strain stem from TLS during replication, either directly at the fork or more likely during subsequent gap-filling behind the fork [8]. In an *umuDC* strain, the induced mutation frequency curve is located well below the *dinBpolB* curve, close to background, confirming that Pol V is involved in both NER-dependent and NER-independent mutagenesis pathways.

### NERiM versus RiM: Kinetics of mutation induction (Fig 2)

**Fig 2:**
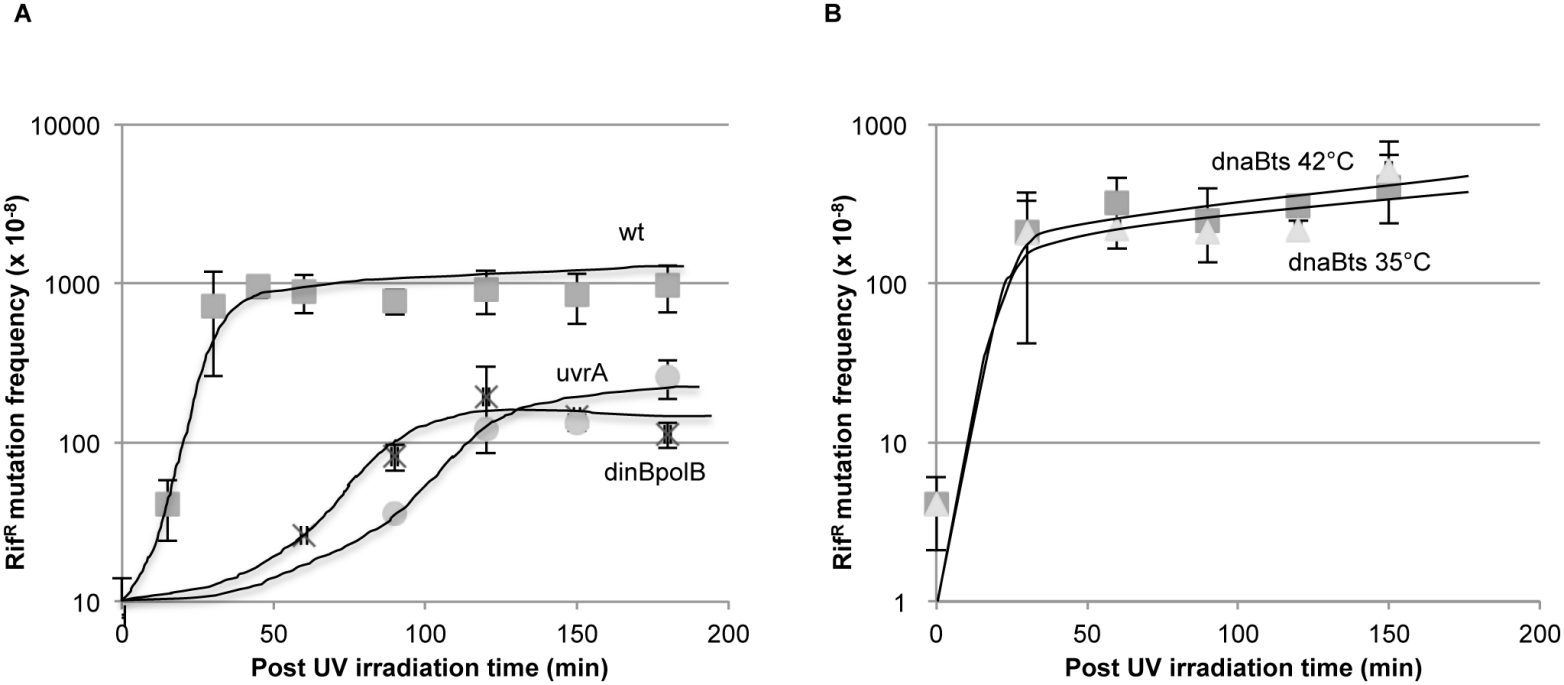
Differential kinetics of mutations induced during NER versus replication. **2A:** Kinetics of rif^R^ mutation fixation in various strains following a single UV dose. The UV dose was chosen so as to affect survival of the various strains to a similar extent i.e. ≈10%: wt ■(squares): 100 J/m^2^, *dinBpolB* X (crosses): 35 J/m^2^, *uvrA* •(dots): 3 J/m^2^. Following irradiation at the indicated UV dose, cells were platted on rif plates at various time points. Rif^R^ mutations accumulate rapidly in the wild-type strain (t_1/2_ ≈ 30 min after irradiation); in contrast the kinetics of appearance of mutants in the *uvrA* and in the *dinBpolB* strains are severely delayed (t_1/2_ ≈ 80–100 min after irradiation). The plateau reached in the *uvrA* and *dinBpolB* strains correspond to ≈ 25–30% and 15–20% of the level reached in the wild-type strain, respectively (see also Fig 1E). **2B**: Kinetics of induction of rif^R^ mutation in a *dnaBts* thermo-sensitive replication mutant irradiated at 90 J/m^2^ [45,46]. At this dose, survival of *dnaBts* strain is 11.5%. The *dnaBts* strain quickly stops replication when shifted at 42°C, the non-permissive temperature. Rif^R^ mutants are induced at similar kinetics at permissive (35°C: Δtriangles) and non-permissive (42°C: ■squares) temperature, implying that most mutations are fixed in a non-replicative manner.

As discussed in the introduction, following UV irradiation, bacterial cells temporarily stop replication to allow removal of most lesions by NER before resuming replication [14–16,23]. We thought to exploit this feature as a way to differentiate mutations that are fixed during repair from those that arise during replication by analyzing the induction of mutations in various strains as a function of time after UV. We thus compared the kinetics of rif^R^ mutation fixation in various strains following a single UV dose. The UV dose was chosen so as to affect survival in the various strains to a similar extent, namely ≈10% *(wild-type*: *100 J/m*^*2*^, *dinBpolB*: *35 J/m*^*2*^, *uvrA*: *3 J/m*^*2*^*)*. While rif^R^ mutations accumulate rapidly in the wild-type strain (t_1/2_ ≈ 30 min after irradiation), the kinetics of appearance of mutations in the *uvrA* and in the *dinBpolB* strains are very significantly delayed (t_1/2_ ≈ 80–100 min after irradiation) (Fig 2A). Mutations fixed during replication appear with a delayed kinetics compared to NER-associated mutations. The plateau reached in the *uvrA* and *dinBpolB* strains corresponds to ≈ 25–30% and 15–20% of the level reached in the wild-type strain, respectively (see also Fig 1E at 10% of survival).

As indicated above, for a wild-type strain a dose of 100J/m^2^ corresponds to a similar level of survival (≈10%) as a dose of 35J/m^2^ administered to a *dinBpolB* strain (Fig 1C). According to our mathematical model (see below), the level of RiM as deduced from the *dinBpolB* strain at 35J/m^2^ corresponds to only ≈15% of the total level of mutagenesis induced at 100J/m^2^ in the wild type strain. This is compatible with the observation that in the wild-type strain most of the induced mutagenesis occurs rapidly via NERiM and that only a small additional increase in mutagenesis appears at longer time points (corresponding to ≈15% of RiM).

To further establish this hypothesis we conducted UV-induced mutagenesis experiments in a thermo-sensitive *dnaB* helicase mutant. These experiments were conducted at a UV dose of 90 J/m^2^ corresponding to about 10% of survival (Fig 2B). In a *dnaBts* strain, known to be a quick-stop replication mutant [45,46], rif^R^ mutants are induced at similar kinetics at both permissive (35°C) and non-permissive (42°C) temperature, implying that most mutations are fixed in a non-replicative manner (Fig 2B).

### A two-hit dose-response curve suggests an “opposing lesion” model for NERiM (Figs 3 and 4)

**Fig 3:**
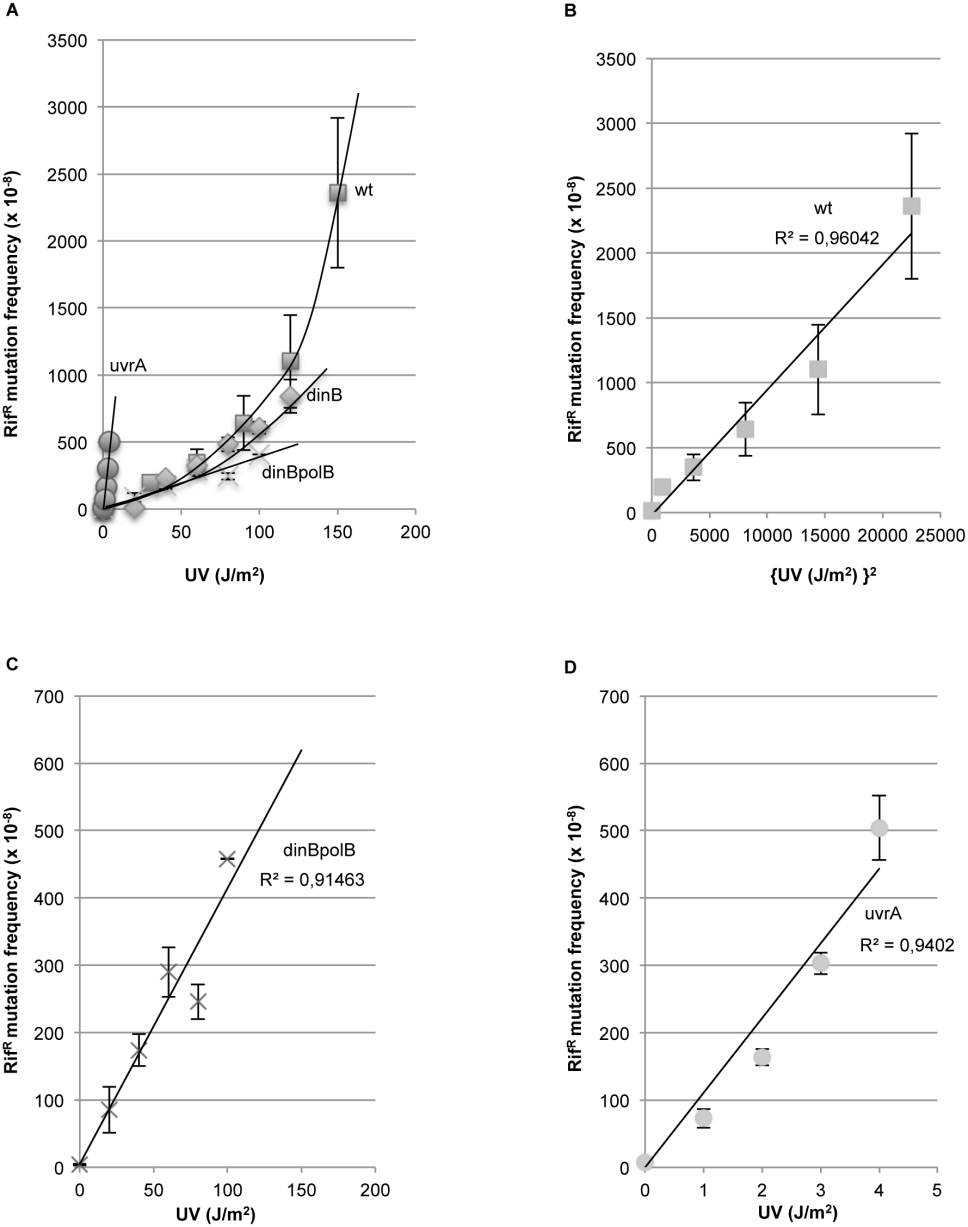
Mutations induced during NER and replication: Two hits versus single hit dose response curves (or quadratic versus linear dose response curves). The induction of rif^R^ mutation frequencies as a function of UV dose in wild-type ■ (squares), *dinBpolB* X (crosses) and *uvrA* •(dots) strains (average values of 5 independent experiments per strain). A: In the wild-type strain, the mutation frequency expressed as a function of UV dose, is clearly non-linear. B. The wild-type data fit a straight line when mutation frequencies are plotted as a function of square dose (regression coefficient = 0.96), strongly suggesting that most mutations arise via a two-hit mechanism such as the processing of closely spaced lesions. C and D: In strains *dinBpolB* (regression coefficient = 0.91) and *uvrA* (regression coefficient = 0.94) the mutation frequency response is linear with UV dose as expected when mutations occur at the fork during replication.

**Fig 4:**
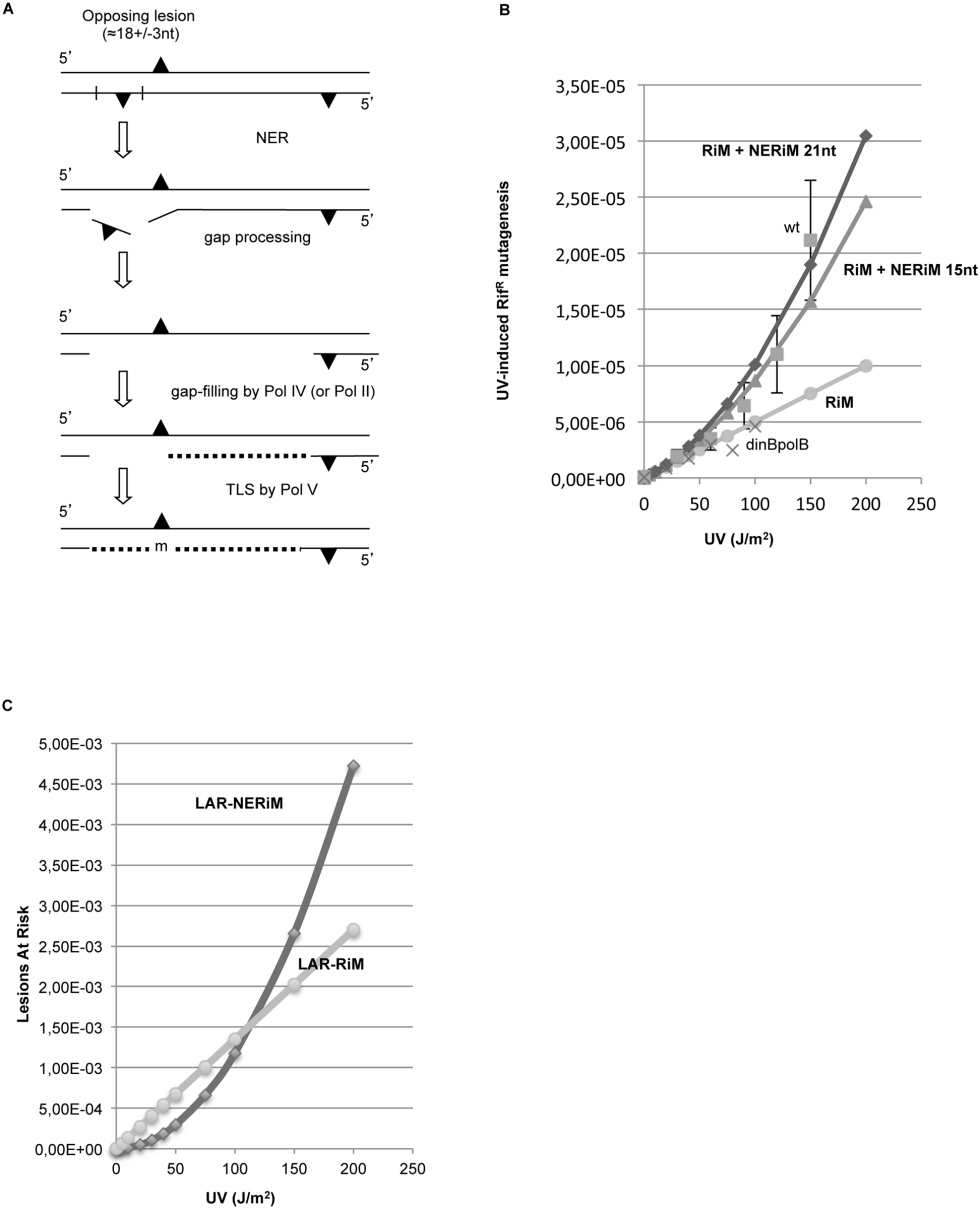
Opposing lesion structure model for NER-induced mutagenesis. **A**: Nucleotide Excision Repair initiates repair at a given lesion via its normal dual incision step creating a 12–13 nt single-stranded gap. At rare occasions a second lesion will be located in the opposite strand within the initial incision gap or in close proximity. In the latter case the second lesion may becomes exposed within the gap following a gap enlargement step possibly triggered by the distortion induced by the second lesion at the double-stranded / single-stranded junction via exonuclease (or helicase) processing. The resulting structure will be referred to as “opposing lesion structure”. Alternatively, gap enlargement may result from the necessity to allow a RecA filament to assemble downstream from the lesion in order to activate Pol V [47]. The gap-filling process requires the action of Pol IV/Pol II. The mutation “m” opposite the lesion site is fixed by Pol V during the lesion bypass step. **B**: Mathematical modeling of UV-induced mutagenesis in a wild-type and *dinBpolB* strains as a function of UV dose (J/m^2^). The RiM line fits properly the *dinBpolB* data points •(dots) (regression coefficient R = 0.91). The wild-type data points ■ (squares) fit appropriately the theoretical curve when the region that defines the opposing lesion zone is set to 18 +/-3 nt (i.e. data points within the 15 and 21 nt range). **C**: Number of lesions “at risk” (LAR) as a function of UV dose (J/m^2^). The straight line and the quadratic curve represent the number of lesions, within the *rpoB* target, at risk for inducing mutations during replication (LAR-RiM) or during NER (LAR-NERiM: opposing lesion zone set to 18nt), respectively (see text). At ≈100J/m2, when the two curves cross, an equal number of mutations will result from replication and NER pathways.

How may UV-induced mutagenesis be triggered during NER? We envision two formal possibilities i) the “faulty NER” model: at low frequency, the NER machinery makes a “mistake” and incises the non-damaged strand thus generating a lesion-containing gap or ii) the “opposing lesion structure” model: a proper NER-mediated repair event occurs at a location where a second lesion resides in close proximity in the complementary strand. In both models, the gap resulting from NER incision contains a lesion that may lead to a mutation during subsequent gap filling. To distinguish between the two models, we analyzed the shape of the induced mutagenesis curve as a function of UV doses. In the wild-type strain, mutation frequency data exhibit a near-quadratic behavior with respect to UV dose (Fig 3A and 3B) while the relationship is linear in both *dinBpolB* (Fig 3C) and *uvrA* (Fig 3D) strains. The quadratic response in the wild-type strain suggests that NERiM results from the processing of opposing lesions (see below). In contrast, the linear relationship of mutation frequency with dose in the *dinBpolB* and *uvrA* strains indicates a single hit model as expected when mutations are fixed during replication (RiM).

We propose the following working model for NERiM (Fig 4A): normally, NER proceeds via excision of a short 12–13 nucleotide-long tract that contains a damaged nucleotide, followed by repair synthesis of the short gap by DNA Pol I. This process is error-free and leads to a spectacular increase in cell survival (Fig 1C). At rare occasions a second lesion will be located in the opposite strand either within the initial incision gap or in close proximity, near the gap junctions. When the second lesion is located within the initial gap, a gap enlargement step may be necessary to allow a RecA filament to assemble downstream from the lesion in order to activate Pol V [47]. If the second lesion is located near the initial gap junction, it may become part of the gap following a gap enlargement step triggered by the distortion induced by the second lesion at the double-stranded / single-stranded junction via exonuclease (or helicase) processing. The resulting structure will be referred to as “opposing lesion structure”. Closely spaced photoproducts in complementary strands occur at high (but physiologically relevant) doses [48]. Published data suggest that opposing UV dimers are substrates for NER incision. Indeed, opposing cyclobutane dimers within the 5’-TTAA sequence are properly incised by UvrABC, in one or the other strand, without double strand break formation [49]. The frequency of opposing lesion structures increases with the square of the UV dose. The present model posits that repair of the opposing lesion structure requires both Pol V at the TLS step across the lesion and Pol IV / Pol II for yet unknown reasons (see below the § about Damaged Primer Elongation) (Fig 4A). However, it does not exclude the potential participation of Pol III in the gap-filling reaction as previously suggested by Bridges and coworkers [50].

This model predicts similar UV-sensitivities for *dinBpolB* and *umuDC* strains, an expectation not met experimentally as a *umuDC* strain is found to be less UV-sensitive than a *dinBpolB* strain (Fig 1C). The higher UV sensitivity of *polBdinB* compared to *umuDC* could be accounted for by the existence of NER-induced gaps that require processing by Pol IV/Pol II but not by Pol V. Such gaps may contain a specific UV lesion that can be bypassed by Pol IV/Pol II without need of Pol V (as discussed above: the putative lesion at position 526). Alternatively, when two lesions are in close proximity in the same strand, incision at one of them may generate a lesion-containing primer that requires Pol IV / Pol II for its extension (see below) but not necessarily Pol V as the gap may not contain a lesion. Such events account for greater UV sensitivity of *dinBpolB* compared to *umuDC* strains.

The precise role of Pol IV (and to a lesser extent of Pol II) during the completion of the gap-filling reaction during NERiM remains a puzzle for which we offer some genetic and biochemical hints (see below). To better define the requirement for Pol IV, we wondered whether its role in NERiM requires binding to the beta-clamp, the replication processivity factor [51]. For this purpose we used a mutant *dinB* gene carrying a five amino acid C-terminal deletion of the consensus beta-clamp binding motif [52]. Compared to a plasmid expressing dinB+, a plasmid expressing the *dinBΔ5* mutant only partially complemented a *dinBpolB* strain (Fig 1A), suggesting that the interaction of Pol IV with the beta-clamp is important for its function in NERiM. Interestingly, Pol kappa, the eukaryotic ortholog of Pol IV was shown to be involved in NER [53]. The model proposed could explain the finding that mouse cells lacking Pol kappa are defective at excising lesions from DNA and filling NER gaps [54].

### Mathematical model for UV-induced mutagenesis during NER and replication (Fig 4B and 4C)

In order to estimate the mutation frequencies induced by UV lesions during NER (NERiM) or replication (RiM) as a function of UV dose (D), we have generated the following mathematical model. RiM and NERiM will be calculated by multiplying the number of lesions subject to either replication or NER by the average rate of conversion of a lesion into a mutation in the rif assay.

The following parameters have been used:

1. Lesion density: It has been determined that a UV dose of 1J/m^2^ generates ≈ 50 lesions per *E*. *coli* genome [55]. For a UV dose D (J/m^2^), the average lesion density “x” per DNA strand per nucleotide is thus x = 25/4,6.10^6^.D = 5,4.10^-6^.D.
2. The main target for rif^R^ mutations (>90%) is a 250 base pair fragment located in the *rpoB* gene; rif^R^ mutants result from specific base substitutions within the *rpoB* gene [36].
3. Rate of conversion of a UV lesion into a rif^R^ mutant during TLS: This rate can be estimated from the slope of the induced mutation dose-response curve in the *uvrA* strain (Fig 3D). A UV dose D = 1J/m^2^ induces ≈ 10^−6^ rif^R^ mutants. Given the relationship x = 5,4.10^-6^.D, where x is the average lesion density per strand per nt, it can be derived that the number of UV lesions formed at 1J/m2 within the double-stranded 250 bp target is equal to 2.250.5,4.10^−6^ = 2700.10^−6^. As shown recently, at the replication fork, only about 10% of the lesions are subject to TLS under SOS-induced conditions while 90% are tolerated by Damage Avoidance [56], it can thus be estimated that it takes ≈ 270. 10^−6^ UV lesions located within the 250 bp target to produce ≈10^−6^ rif^R^ mutants via TLS. Thus ≈ 270 lesions on average produce one rif^R^ mutant. A lesion-to-mutation conversion factor of 1/270 = 0,0037 will thus be used to estimate both the frequencies of replication- and NER- induced mutations.
4. In a NER-proficient strain, we estimate that ≈ 95% of UV lesions are removed by NER, while the remaining ≈5% lesions are processed during replication. This figure derives from the following experimental data: in a *dinBpolB* strain (Fig 3C), only replicative mutants are monitored since the process of NERiM is abolished. As expected for mutations occurring at the replication fork, the mutation frequencies in the NER-deficient (*uvrA*) and NER-proficient (*dinBpolB*) strains vary linearly with UV dose (Fig 3D and 3C). The slope in the uvrA strain (Fig 3D) is about 20–25 fold steeper than in the *dinBpolB* strain (Fig 3C), suggesting that the mutation frequency in the NER-proficient *dinBpolB* strain can be accounted for by 5% of non-repaired lesions. A similar figure is found when comparing the mutant frequency induced by a single T(6–4)T adduct in wild type (0.4%) and uvrA (8.9%) strains [57].

#### Mutations occurring during replication (RiM)

RiM = number of lesions subject to replication times the rate of conversion into a mutation by TLS. As discussed above, we estimate that ≈ 5% of the initially formed lesions escape NER and will thus be subject to replication fork processing. On the other hand, only ≈ 10% of these lesions will be processed by TLS [56]. The number of lesions subject to TLS within the 250 bp target is thus equal to 2 .250. 0,1. 0,05. 5,4.10^−6^.D = 13,5. 10^-6^D; the conversion factor being 0,0037, it follows that RiM = 5.0.10^-8^D. (lesions exposed to RiM = 1,35. 10^-5^D)

#### Mutations occurring during repair (NERiM)

NERiM = number of lesions at risk times their rate of conversion into a mutation by TLS. We hypothesize that NERiM occurs when, during the course of a regular repair event, a second lesion is located in the opposite strand in close proximity (defined as opposing lesions, Fig 4A). Our experimental data fit best the theoretical curve if the second lesion resides within a patch of ≈18+/-3 nt located in the strand opposite the initial incision tract. As mentioned above, our calculation takes into account that 95% of the initially formed lesions are processed by NER. The probability of forming an opposing lesion structure located within a 250 nt target is 250. 0,95. 5,4.10^−6^.D times the probability of a second lesion being located in a 18 +/-3 nt long region: 18. 0,95. 5,4.10^−6^.D = 11,8. 10^-8^D^2^; the conversion factor being 0,0037, it follows that NERiM = 4,38.10^−10^. D^2^. (lesions exposed to NERiM = 1,18. 10^-7^D^2^).

We plotted the above-derived equations for RiM and RiM+NERiM as a function of D (Fig 4B) and superimposed the actual data points determined in the *dinBpolB* and wild-type strains. The RiM line fits well the *dinBpolB* data points (regression coefficient R = 0.91) (Fig 4B). It should be noted that the mathematical model for NERiM is derived, essentially *ab initio*, with only one adjusted parameter, namely the size of the *opposing lesion zone* that fits best the experimental data when set at 18 +/-3 nt. Indeed, the two curves corresponding to *opposing lesion zone* values of 15 and 21 nt appear to frame satisfactorily the wild-type data points (Fig 4B).

Respective contributions of RiM and NERiM at low UV doses: It can be estimated that at ≈ 40J/m^2^, a dose that only moderately affects survival in a wild-type strain (≈60% of survival), the mutation frequency induced by UV during NER corresponds to 50% of the mutation frequency induced at the fork (Fig 4B). While the number of opposing lesions involved in NERiM are less abundant at low UV dose, they become equal to the number of lesions processed at the fork at about 100J/m^2^ (Fig 4C).

### How general is NERiM? (Fig 5)

**Fig 5:**
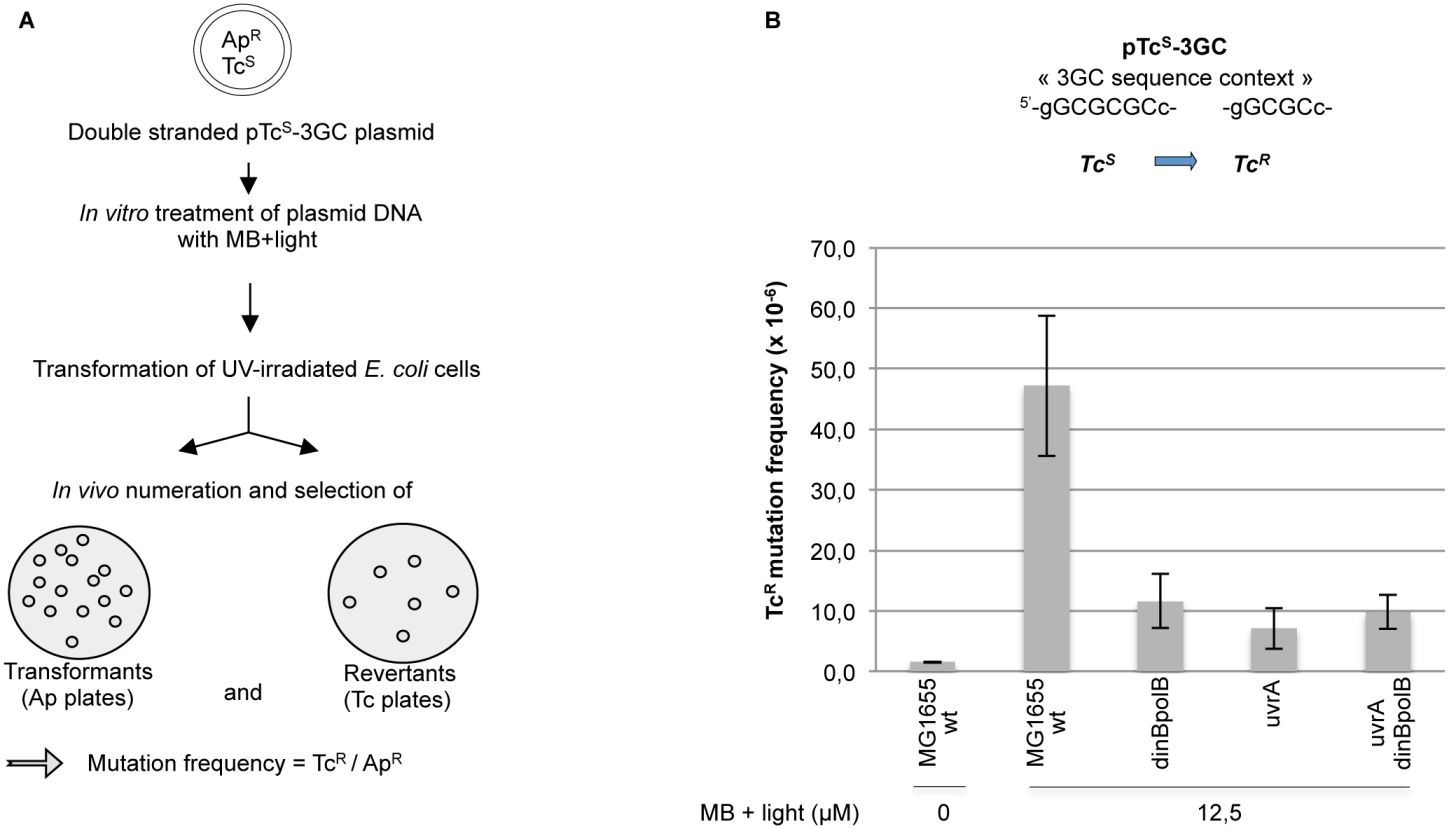
Plasmid-based assay to show NERiM for oxidative lesions. How general is NERiM? The involvement of NER in induced mutagenesis was investigated by implementation of a plasmid-borne mutation assay using a different mutagenic treatment, namely oxidative damage induced by treating plasmid DNA with Methylene Blue and visible light (MB+ light). **A**: Experimental outline: the assay involves a pBR322-derived plasmid that contains a +2 frameshift mutation within its tetracycline resistance gene [58]. The assay was shown to monitor true -2 frameshift reversions that restore tetracycline resistacrossnce [59,60]. Plasmid DNA is randomly damaged *in vitro* with MB+ light treatment and introduced into bacteria by transformation. Cells are plated on tetracycline and ampicillin plates to determine the Tet^S^-> Tet^R^ reversion frequency. **B**: cells are treated by UV irradiation prior to plasmid transformation in order to induce their SOS response. The UV dose is chosen so as to yield a survival of 10–20% (wild-type: 95 J/m^2^; dinBpolB: 45 J/m^2^; uvrA: 5 J/m^2^ and uvrAdinBpolB: 4 J/m^2^). The average and standard deviation of four to six independent determinations are plotted for each strain. Introduction, into wild-type cells, of plasmid DNA treated with MB+light robustly increases the -2 frameshift mutation frequency by two to three orders of magnitude above untreated control plasmid. The mutagenic response is reduced ≈5-fold when the (MB+light) treated plasmid is introduced into either *uvrA*, *dinBpolB* or *uvrAdinBpolB* strains.

We wondered whether the NER-induced mutation pathway described above and established within the frame of a chromosomal mutation assay (rif^R^) using UV lesions as the mutagen could be generalized to another mutagen and another assay. For this purpose we implemented a plasmid-borne reversion assay using as a mutagen oxidative lesions induced by the *in vitro* treatment of plasmid DNA with methylene blue plus visible light (MB+light) and its subsequent introduction in bacterial cells for mutagenesis monitoring. This mutation assay involves the reversion of a tetracycline sensitive to a tetracycline resistant allele carried by a pBR322-derived plasmid [58]. Plasmid DNA is randomly damaged *in vitro* with (MB+ light) and introduced into bacteria by transformation. The assay was shown to be highly specific for monitoring only true -2 frameshift revertants to tetracycline resistance [59]. This assay is highly sensitive as the level of induced mutagenesis is two to three orders of magnitude above the background level reached with untreated control plasmid [60]. Cells are plated on tetracycline and ampicillin plates to determine the Tet^S^-> Tet^R^ mutation frequency. The (MB+light) treatment, that induces a variety of oxidative lesions, was previously shown to trigger -2 frameshifts in an SOS-dependent way [60]; although the chemical nature of the culprit lesion is unknown, it was shown not to be 8-oxo-dG [60]. Introduction into SOS-induced wild-type cells of plasmid DNA treated with (MB+light) robustly increases the -2 frameshift mutation frequency by two to three orders of magnitude above untreated control plasmid (Fig 5A and 5B). The mutagenic response is reduced ≈5-fold when the (MB+light) treated plasmid is introduced into either *uvrA*, *dinBpolB* or *uvrAdinBpolB* strains (Fig 5B). In a *umuDC* strain the induced-mutation frequency is fully abrogated [61]. These data, obtained within the frame of a plasmid-borne mutation assay, fully recapitulate the key genetic requirements of NERiM as established above with the chromosomal rif^R^ assay. It thus further generalizes NERiM to different mutagenic treatments and to all types of point mutations, base substitutions and frameshift mutations. Moreover, the plasmid assay could be implemented in all strains at the same lesion density, thus validating the NERiM pathway established at doses leading to equal survival but with different lesion densities in the chromosomal context (see further discussion in Conclusion §).

### UV-induced mutagenesis in stationary phase bacteria (Fig 6)

**Fig 6:**
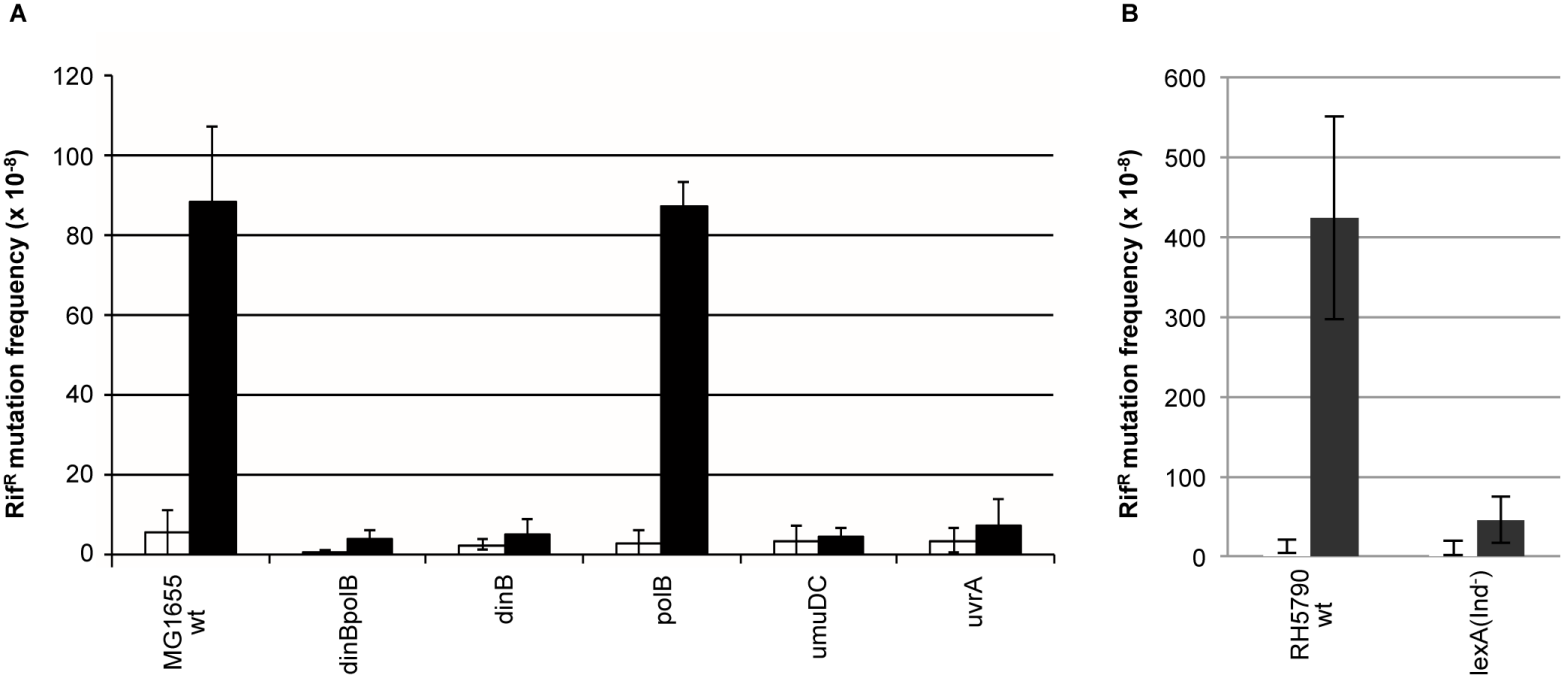
Occurrence of NERiM in stationary phase *E*. *coli* cells. When irradiated at 200 J/m^2^ in stationary phase the survival of the wt and uvrA strains were equal to 75% and 63%, respectively. **A**: Induction of rif^R^ mutations by UV-light at a dose of 200 J/m^2^ (black bar) or in the absence of irradiation (white bar) in stationary phase *E*. *coli* cells. **B**: suppression of UV-induced mutagenesis in stationary *E*. *coli* cells that are defective in SOS induction: *lexA*(Ind) strain.

Next, we wondered about the biological significance of NERiM, a mutation pathway that is coupled to NER instead of being linked to replication. A NER-dependent pathway appears to be advantageous to stationary phase cells in order to adapt to genotoxic conditions. To investigate directly this possibility we UV-irradiated stationary-phase bacteria contained in 1 to 3 day-old *E*. *coli* colonies (doses 200–400 J/m^2^) and further incubated the colonies for periods ranging between 1 to 3 days. At the end of the incubation period, the level of rif^R^ mutants was determined by suspending individual colonies in liquid medium and plating adequate dilutions on LB or LB + rifampicin containing plates. In wild-type bacteria, we observe a 30 to 50-fold increase in rif^R^ mutagenesis in irradiated compared to non-irradiated control colonies. Strikingly, induced mutagenesis in these stationary phase *E*. *coli* cells was entirely dependent upon a functional excision repair system (i.e. absence of induced mutagenesis in a *uvrA* strain) suggesting that mutagenesis arose during NER (Fig 6A). In order to determine which DNA polymerase was involved in the generation of these UV-induced mutations, we examined strains defective in each of the specialized DNA polymerases, Pol II, Pol IV and Pol V. In addition to the expected involvement of Pol V, UV-induced mutagenesis in stationary phase cells was totally dependent upon a functional Pol IV gene, while Pol II had essentially no effect. Thus the genetic control of stationary phase mutagenesis induced by UV irradiation is similar to NERiM except for the involvement of Pol II, an observation that remains unexplained. We also found that UV-induced mutagenesis in stationary phase cells is suppressed in a strain that cannot induce its SOS system (*lexA*(Ind)- strain) (Fig 6B). It has also been reported that spontaneous mutagenesis in resting bacterial populations is controlled by the SOS-response in a cAMP-dependent way [62]. Using an F plasmid based reversion assay, it was shown that spontaneous -1 frameshift mutations in short runs depend upon Pol IV and recombination proteins in stationary phase cells [63]. Detection of lesions in stationary phase may be initiated during transcription by the Transcription Coupled Repair factor Mfd as suggested by a recent study on stationary phase mutagenesis in *B*. *subtilis* [64]. In *B*. *subtilis* it was also found that stationary-phase mutagenesis involves the *yqjH* gene that encodes a Pol IV homolog [65].

### A novel role for specialized DNA polymerases? Damaged Primer Extension reaction (DPE)

Our present working model for NER-induced mutations involving opposing lesions (Fig 4A) raises two major questions: how are the extended gaps formed and how are they subsequently filled-in? We hypothesize the involvement of an exonuclease activity that will use as its substrate a normal NER gap formed at opposing lesion structures (as defined above, Fig 4A). Similarly, in *S*. *cerevisiae* it was shown that the occasional processing of normal NER incision intermediates by Exo1 leads to extended gaps that are essential in checkpoint activation in non-dividing cells [66,67]. The rare occurrence of large NER gaps has previously been described in *E*. *coli* [68,69]. Indeed, large NER gaps (≈1500 bases) were found at about 1% the frequency of the normal NER gaps for UV doses in the 60J/m^2^ range [68,69]. The enzymatic activities involved in gap enlargement in *E*. *coli* are unknown. *E*. *coli* possesses numerous exonucleases preventing us to unequivocally identify the responsible exonuclease(s). Degradation by exonucleases of DNA templates containing chemically modified nucleotides has revealed that the degradation of lesion-containing templates is blocked at or near the lesion sites [70,71]. If this is the case, the subsequent gap-filling reaction requires a DNA polymerase able to extend a primer containing a lesion in the vicinity of its 3’-end (Fig 4A). In order to support such a model, the ability of *E*. *coli*’s DNA polymerases to extend damaged primer extremities *in vitro* was analysed. Primers containing either a TT-CPD or a T(6–4)T photoproduct at their 3’-end (S3 Fig) were annealed to a single-stranded circular DNA substrate (S4 Fig). These primer / templates were subsequently incubated in the presence of the β-clamp, the clamp loader, RecA and SSB proteins before addition of a given DNA polymerase [12]. For sake of comparison, an equivalent amount of DNA pol units of Pol I, II, III*, IV and V were added to these primer template substrates although this does not reflect the respective amounts of the different DNA polymerases in *E*. *coli* cells. Following a 30 minutes incubation period, replication products were analysed by PAGE. The data show that for the TT-CPD containing primer, Pol IV (and Pol II) show significantly greater extension capacities compared to Pol I and Pol V, while Pol III* is intermediate (S4 Fig). With respect to the T(6–4)T bearing primer, Pol II exhibits the greatest extension potential (S4 Fig). The lack of extension activity by Pol I is in good agreement with our *in vivo* data showing that NERiM is fully proficient in the *polA1* strain (Fig 1B). In good agreement with genetic data, Damaged Primer Extension of TT-CPD and T(6–4)T lesions by Pol IV and Pol II, rely on the presence of the β-clamp (S5 Fig) (Fig 1A). These data support the idea that Pol IV / Pol II may be involved in NERiM by virtue of their capacity to extend primers that contain lesions at their 3’-extremity (S6 Fig).

## Conclusion

In recent years it was established that most induced point mutations occur as a consequence of translesion synthesis (TLS) a process during which specialized DNA polymerases copy a DNA template that contains a damaged base. The process of TLS and of mutagenesis was primarily thought to occur during the course of replication either directly at the fork (“on the fly”) or during subsequent gap-filling (see review [8]). *E*. *coli* possesses three DNA polymerases (Pol II, Pol IV and Pol V), under the control of the SOS response, all three of which are potentially involved in TLS and mutagenesis, depending upon the chemical nature of the lesion and the local sequence context [72]. In the present paper we characterize a mutagenesis pathway due to Translesion Synthesis at lesions that become exposed in gaps during the process of Nucleotide Excision Repair (NER).

The present paper describes the genetic requirements of a mutagenesis pathway associated with NER. While NER is clearly an important repair pathway that removes the vast majority of DNA lesions in an error-free way, it encompasses a mutagenic sub-pathway to which we refer to as NERiM, for NER-induced Mutagenesis to distinguish it from the mutagenesis pathway that occurs during Replication (RiM).

A major conceptual concern that arose during the course of the present work was to find a way to meaningfully compare induced mutation frequencies among strains that exhibit dramatically different UV sensitivities? Should induced mutation frequencies be compared at equal UV doses or at equal UV survival? The three major strains under consideration are wild type, *dinBpolB* and *uvrA*. At a UV dose of 5 J/m^2^, the survival of the two first strains is nearly 100% while it is only ≈1% for the *uvrA* strain. On the other hand, a similar level of survival (i.e. ≈ 10%) is achieved at UV doses equal to 90, 35 and 3 J/m^2^ for wild type, *dinBpolB* and *uvrA*, respectively. We decided to compare induced mutation frequencies as a function of survival. Under these conditions, in *dinBpolB* and *uvrA* strains, the induced mutation frequencies (Figs 1A and 5B) and their kinetics of induction (Fig 2A) turned out to be similar, suggesting that the vastly different lesion density (compare 35 to 3 J/m^2^) did not affect the mutagenic outcome in these two strains. Conversely, the large difference in induced mutation frequencies (Figs 1A and 5B) and in their kinetics of induction (Fig 2A) between a wild type strain and *dinBpolB* strains, despite the relatively modest difference in lesion density (compare 35 to 90 J/m^2^), points to the existence of a specific *dinBpolB* mutation sub-pathway. Taken together, these observations tend to validate the choice of comparing induced mutation frequencies at equal doses of survival. Our concern about establishing the chromosomal NERiM pathway on experiments comparing strains at equal UV survival, and thus at different lesion densities, was essentially released when the genetics of the NERiM pathway (i.e. dependence upon *polBdinB* and *uvrA*) could be recapitulated using plasmid-based assays (Fig 5) that were performed at equal lesion densities (MB+light) in all strains.

### The major features of NERiM are

#### 1. NERiM involves the NER incision machinery and a specific set of DNA polymerases

NERiM requires the early NER recognition/incision genes (*uvrA*, *uvrC*) and Pol IV (*dinB*) and to a lesser extent Pol II (*polB*) for the latter re-synthesis steps. Pol IV, encoded by the *dinB* gene product, exhibits numerous phenotypes among which i) its capacity to act as a TLS polymerase specifically at various DNA adducts located in the minor groove of the DNA helix such as benzo(a)pyrene-, nitrofurazone-N2-guanine adducts as well as adducts formed by alkylating agents at N3-methyladenine ([72–74]) ii) *dinB* is involved in long term survival and evolutionary fitness under physiological expression levels [75], but when over-expressed Pol IV interferes with chromosomal replication inducing -1 frameshift mutations [41,76] and leads to cellular toxicity [77,78]. These activities are mediated by β-clamp interactions [51,79,80] and III*) its involvement in adaptive mutagenesis [81]. Interestingly, the present work describes a novel phenotype of *dinB*, in combination with *polB*, in the context of NER. Indeed, a *dinBpolB* double mutant strain exhibits reduced UV-survival and reduced UV-mutagenesis. This leads to define a *dinBpolB* pathway for both survival and mutagenesis that was shown to be epistatic to the NER pathway (Fig 1D and 1E). In addition to Pol IV / Pol II, NERiM also requires the *umuDC* encoded Pol V for TLS but not Pol I (*polA*) that is normally involved in NER gap re-synthesis (Fig 1A and 1B).

#### 2. NERiM occurs in non-dividing cells as well as in growing cultures

In a wild-type strain, UV-induced mutations are fixed between 10 and 40min, a time frame of active NER and of strongly impaired replication (Fig 2A). In contrast, when NER is inactivated, UV-induced mutations appear at a later time frame (80–150 min) that coincides with full recovery of replication (Fig 2A). Further evidence for the existence of replication-independent mutations was obtained in a strain with a thermo-sensitive replication-defective mutation (*dnaBts*). Indeed, the kinetics of accumulation of UV-induced mutations is similar at both non-permissive (i.e. in the absence of replication) and permissive temperatures (Fig 2B). We also show that NERiM operates in stationary phase cells with similar genetic requirements (Fig 6).

#### 3. NERiM exhibits a quadratic dose-response curve suggesting the involvement of opposing lesions (Figs 3 and 4)

Mutations that occur in strains where NERiM is inactivated (in *uvrA* or *dinBpolB* strains) exhibit a linear dose-response relationship as expected when a lesion is converted to a mutation at the replication fork (Fig 3C and 3D). In contrast, mutations produced when NER is functional, i.e. in a wild type strain, exhibit a dose-response curve with quadratic characteristics (two-hit kinetics) (Fig 3A and 3B). As discussed above, the square dose response suggests the involvement of closely spaced lesions shown, for example, to be located on opposite strands (Fig 4A). Fitting an *ab initio* mathematical model to the experimental data hints at the involvement of two lesions being located within a critical zone of 18 +/-3 nt (Fig 4B).

#### 4. NERiM is not restricted to chromosomes, it also operates in plasmids

Using a plasmid-based frameshift reversion assay and a mutagenic treatment with an oxidative agent instead of UV light we show that NERiM functions under a similar set of genetic requirements (Fig 5).

### Unresolved questions and future perspectives

#### How are opposing lesion structures generated?

Mathematical modeling suggests that opposing lesion structures are generated when an initial NER event takes place at a lesion site where a second lesion resides in the complementary strand within a critical ≈18 nt distance. The second lesion can thus be located either directly within the initial 12 nt incision gap [49] or situated close to one of the gap junctions. In any case, repair of opposing lesion structures is likely to involve gap-processing steps such as gap enlargement by exonucleases and/or helicases. Genetic data presented here suggest that opposing lesion structures are toxic intermediates unless processed by the *dinB* and *polB* gene products. Additionally, processing of these toxic intermediates by Pol IV / Pol II also involves Pol V and leads to NER-induced mutagenesis. The biochemical basis for the requirement of Pol IV and/or Pol II has tentatively been ascribed to their properties to extend damage containing primers but these steps will need further characterizations. Conceptually, closely spaced lesions in opposite strands pose a challenge to repair analogous to inter-strand cross-links (ICL); after incision of one strand the opposite strand contains another lesion. It is of interest to note that an ICL repair pathway involving *polB* has been described in *E*. *coli* [82].

#### Potential importance of NERiM as a contributor to genetic diversity in non-replicating cells

Under stationary phase conditions, induced mutagenesis strictly depends upon a functional NER system and upon Pol V and Pol IV (but not Pol II) thus essentially mimicking the NERiM pathway described under exponential phase conditions. We would like to stress that at low lesion density, even though opposing lesion structures may be rare, NERiM can significantly contribute to mutagenesis. It can be estimated that the intrinsic rate of conversion of a lesion into a mutation is 10–100 fold higher when present within an opposing lesion structure compared to it being present at the fork. Indeed, at the fork, homologous recombination with the sister chromatid ensure most lesion tolerance events strongly preventing mutagenesis [56,83,84]. Outside the context of replication, when the lesion is located in a gap generated by NER, its repair most likely involves a TLS event; indeed, repair of the gap by recombination with a homologous chromosomes is highly dis-favored compared to recombination with the nearby sister chromatid as in the event of a replication fork associated event.

The present paper highlights the existence of a critical NER intermediate (generated at closely spaced lesions in opposite strands), the so-called opposing lesion structure; repair of this intermediate requires the *dinBpolB* pathway. Lack of repair of this intermediate is highly toxic while its repair is mutagenic. We would like to suggest that mutations resulting from this pathway raise an elegant possibility for on-going evolution in the absence of replication in microorganisms. By the same token, occurrence of mutagenesis in resting bacteria, may represent a novel mechanism for the acquisition of antibiotic resistance. Whether a similar pathway operates in post-mitotic cells in tissues needs to be investigated.

### Experimental procedures

#### UV-survival curves

All survival experiments are conducted under in-actinic light to prevent photo-reversal of UV lesions. An exponentially growing cell culture (OD_700 nm_ = 0,5–0,6) is re-suspended in MgSO_4_ and irradiated at various UV doses (254 nm) monitored by a UV dosimeter (Vilber Lourmat VLX-3.W). Following UV irradiation, adequate dilutions are plated on LB agar to determine the surviving fraction.

#### UV-induced rifampicin resistance mutagenesis assay

An exponentially growing cell culture (OD_700 nm_ = 0,5–0,6) is re-suspended in MgSO_4_ and irradiated at a given UV dose. All operations are conducted under in-actinic light conditions to prevent photo-reversal of UV lesions. Immediately following UV irradiation, an adequate dilution is plated on LB agar for the determination of survival. The irradiated cell suspension is diluted 20-fold in fresh LB medium and incubated at 37°C to allow the expression of UV-induced mutations. After a standard 6h incubation time (unless specified otherwise), the undiluted culture is plated on Rif plates (50μg/ml) to determine the number of UV-induced Rif^R^ mutants. A proper dilution of the culture is plated on LB to determine the number of viable cells. The mutation frequency is expressed as the number of Rif^R^ colonies per 10^8^ cells.

#### Stationary phase UV-induced mutation assay

Overnight bacterial cultures (≈ 10^9^ cells/ml) were diluted 10^4^-fold in LB. Ten μl droplets of this cell suspension were spotted of nitrocellulose filters (Hybond-N+ Amersham, positively charged nylon transfer membrane RPN82B, 0,45μm) laid over fresh LB plates. Plates were incubated at 37°C for 48h. The resulting stationary phase colonies were irradiated by UV light at 200 J/m^2^ or mock irradiated and further incubated for an additional 24h at 37°C. At that time, the Rif^R^ mutation frequency was determined by re-suspending colonies in 1 ml of MgSO_4_ 10 mM. For each mutation frequency determination three individual colonies were used. Each suspended colony was platted on Rif plates (50μg/ml) to determine the number of UV-induced Rif^R^ mutants, while appropriate dilutions were plated on LB to determine the number of viable cells. The mutation frequency is expressed as the number of Rif^R^ colonies per 10^8^ cells.

#### Plasmid-based mutation assay with methylene blue and visible light

Plasmid DNA was treated with methylene blue plus visible light as described [60]. Briefly,10*μ*g of plasmid DNA (CsCl grade, 100 ng/*μ*L in 10 mM Tris (pH 8.0), 1 mM EDTA, and 10 mM MgCl2] in a 96-well microtiter plate placed on ice is exposed to white light after addition of 10 *μ*L of a fresh methylene blue solution (0, 12.5 *μ*M). Light, filtered by a 2 mm thick water layer, is provided for 15 min by a 100 W bulb positioned at a distance of 11 cm above the sample. After irradiation, DNA was ethanol-precipitated 3–4 times and re-suspended in TE buffer [10 mM Tris (pH 8.0), 1 mM EDTA]. Control DNAs were treated as described above, but light was omitted.

## Supporting information

S1 Text Additional experimental procedures, such as rif^R^ mutant sequencing and damaged primer elongation protocols, are described. (DOCX)

S1 Fig Rif^R^ UV-induced mutation spectra in wild type and *dinBpolB* strains (related to Fig 1). UV-induced mutation spectra in the *rpoB* gene leading to rifampicin resistance in the wild-type (**A**) and *dinBpolB* (**B**) strains. Each mutant is represented as a rectangle located either above or below the *rpoB* gene fragment (codons 500–575) depending on the sequence context to match the known preference of UV light to produce lesions at di-pyrimidine sites. The individual base substitutions are color-coded as indicated.
(PDF)

S2 Fig UV-induced mutagenesis. Epistasis of *uvrC*, but not of its homolog *cho* to *dinBpolB*. Rif^R^ mutation frequencies were determined in various strains in response to UV irradiation. All strains are constructed in the MG1655 background. To account for the intrinsic differences in UV sensitivity among strains, we compared UV doses leading to similar levels of survival (as in Fig 1A): grey bars correspond to UV doses leading to survival levels ranging between 5–15%, for black bars survival levels range between 1–5% survival. It should be stressed that at these UV doses, the SOS response is fully induced in all strains. White bars represent the level of spontaneous mutation frequency, i.e. no UV irradiation. Average values and standard deviations are plotted for three or more independent experiments per strain.
(PDF)

S3 Fig Preparation of damaged-primers. In order to get some insight into the specific involvement of Pol IV / Pol II in the gap-filling step during NER-iM, the ability of purified DNA polymerases to elongate primers containing lesions at their 3’-end was investigated. For this purpose, we prepared primers containing lesions at their 3’-end *in vitro*. **A:** Preparation of CPD or T(6–4)T containing primers is outlined. The underlined TT in the 33-mer oligo contains either a TT CPD or T(6–4)T. **B:** The 33-mer oligo or the 33-mer oligo annealed to a 2.7 kb ss-circular DNA are incubated with either Pol III* or Exo III (NEB) for 10 min at 30°C. **C**: Reaction products from B were analysed by PAGE. Lanes 27 TT and 33 TT are control oligos showing the migration of the 27-mer and 33-mer, respectively. The exonuclease associated with Pol III* clearly generates 27-mer CPD and 6–4 oligos from both substrates. On the other hand, Exo III predominantly generates 28-mer CPD oligo (not 27-mer CPD), while it can convert 28-mer 6–4 oligo into 27-mer.
(PDF)

S4 Fig Damaged-primer elongation assays (related to Fig 4). Damaged-Primer Elongation (DPE) by *E*. *coli* DNA polymerases *in vitro*: **A:** Experimental outline for ability of purified DNA polymerases to elongate a primer containing a UV-lesion at its 3’ terminus: The 27-mer CPD or T(6–4)T containing primers (TT CPD or T(6–4)T at the underlined TT sequence), prepared by Pol III* treatment as described in S3 Fig, was annealed on ss-circular DNA. The reaction mixture contains 50 nM β-clamp, 10 nM γ-complex, 10 nM SSB, 2 mM RecA and 2 nM template primer. The 5’ end of the primer is radio-labeled; RecA forms a nucleoprotein filament that is essential for supporting polymerase activity of Pol V [1]. The mixture is incubated for 10 min at 30°C. A given DNA polymerase is added and the mixture is incubated for 30 min at 30°C as indicated. The following amounts of added polymerase correspond to comparable polymerase activity on normal template primer: 5 x 10^−4^ units/μl Pol I KF (USB), 1 nM PolII, 2 nM PolIII*, 4 nM Pol IV or 100 nM Pol V. Finally the reaction product is digested by a restriction endonuclease before PAGE analysis. Detailed procedures have been described previously [1,2]. **B**: Elongation products analyzed by PAGE following incubation with Pol I to Pol V; “–” represents the control with no addition of polymerase. The abundance of end products generated by the *Hin*d III cleavage relative to Pol I are: for CPD, 1.0 (I), 4.3 (II), 2.5 (III*), 2.8 or 12 (if adding 28/29-mer intermediates) (IV), 1.1 (V); for 6–4, 1.0 (I), > 9.2 (II), 2.8 (III*), 1.4 (IV), 3.8 (V).
(PDF)

S5 Fig The β-clamp strongly stimulates elongation. The assays were implemented similarly as described in S4 Fig. In addition to 27-mer CPD and 6–4 primers, a 28-mer CPD primer, that was prepared by Exo III treatment as described in S3 Fig, was also tested. From S3 Fig, the best candidates for CPD and 6–4 primer elongations appear to be Pol IV and Pol II, respectively. We focused these Pols for analysing the effect of the β-clamp. **A:** The presence of the β-clamp is required for efficient 27-mer CPD primer elongation by Pol IV. The β-clamp strongly stimulates elongation by Pol II of the 27-mer T(6–4)T primer. In both cases, while Pol III* mediates β-clamp loading on template DNA, it apparently decreases the efficiency of Pol IV and II elongation product formation. B: In contrast to the elongation of the 27-mer CPD primer, Pol IV is able to elongate the 28-mer CPD primer stepwise even in the absence of the β-clamp. Pol III* again shows negative impact on Pol IV-mediated elongation.
(PDF)

S6 Fig Model for damaged primer elongation. Possible roles of Pol II/IV in the context of UV-induced lesions.
(PDF)
