# Supplementary material for: Processing closely spaced lesions during Nucleotide Excision Repair triggers mutagenesis in E. coli
Source: PLoS Genet. 2017 Jul 7;13(7):e1006881. doi: 10.1371/journal.pgen.1006881 (PMC5521853; doi:10.1371/journal.pgen.1006881)
Supplement: S1 Text — (DOCX) [file pgen.1006881.s001.docx]

**Supplemental experimental procedures**:

1. Rif^R^ Mutation spectra: *rpoB* gene sequencing. The target gene for rif^R^ mutants is the chromosomal *rpoB* gene. Over 90% of rif^R^ mutants are located between codons 500-575 of the *rpoB* gene [3]. The host cells, MG1655 wild-type and MG1655 *dinBpolB* bacteria, were UV-irradiated at 105 and 50 J/m^2^, respectively. For each strain, about 50 individual rif^R^ mutant colonies were isolated and sequenced as described in the main text. Genomic DNA from individual rif^R^ colonies is prepared as follows: an overnight culture is resuspended in TE (Tris-EDTA, pH8), 0,5% SDS, 0,3 mg/ml proteinase K and incubated 1 h at 37°C. After addition of 0,65 M NaCl and 1/10 of the volume CTAB/NaCl (Cetyltrimethyl ammonium bromide 10%) solution, incubation is performed 10 min at 65°C. The DNA is re-suspended in TE buffer after a phenol/chloroform extraction and isopropanol precipitation. The PCR primers 5’ GTCTCTGGGCGATCTGGATA 3’ and 5’ CGTCAGTTACAACACCGTCG 3’ used for sequencing, the genomic fragment located between codons 500-575 of the *rpoB* gene. Sequencing was performed by Genome Express, Grenoble (France).

2. Damaged-Primer Elongation (DPE) protocol: First, we constructed 33-mer oligomers containing an internal TT-CPD or T(6-4)T) photoproduct by ligating a 20-mer: 5’-CCATGATTACGAATTCAGTC to a 13-mer 5'-GCAAGTTAACACG containing the UV lesion (TT-CPD or T(6-4)T) at the underlined TT using the following scaffold oligonucleotide (5’-GACGGCCAGT GCCAAGCTTA GTCCGTGTTA ACTTGCGACT GAATTCGTAA TCATGGTCAT AGCTG) (Fig. S3). Following PAGE purification, the 33-mers (100 nM) were incubated with Pol III* (96nM) at 30°C for 2 hours in the absence of dNTPs. The proofreading exonuclease of Pol III* degrades the 33-mer oligomers until reaching UV-lesion, thus producing a 27-mer with a terminal lesion (TT-CPD or T(6-4)T)) at the 3’-end: 5’-CCATGATTACGAATTCAGTCGCAAGTT. These primers were annealed to single-stranded circular plasmids as described previously [2]. The reaction mixture contains 50 nM β-clamp, 10 nM γ-complex, 10 nM SSB, 2 mM RecA, and 2 nM template primer; the 5’ terminus of the primer is radio-labeled; RecA forms a single-stranded DNA filament that activates Pol V [1](Fig. S4). The mixture is incubated for 10 min at 30°C. A polymerase (5 x 10^–4^ units/µl Pol I KF (USB), 1 nM Pol II, 2 nM Pol III*, 4 nM Pol IV or 100 nM Pol V; these polymerase concentrations exhibit comparable polymerase activity on normal template primer) is added and it is incubated for 30 min at 30°C. Finally the reaction product is digested by a restriction endonuclease for easily visualizing the elongated products (Fig. S4B, Fig. S5). Detailed procedure are fully described in previous papers [1,2].

1. Fujii S, Gasser V, Fuchs RP. The biochemical requirements of DNA polymerase V-mediated translesion synthesis revisited. J Mol Biol. 2004;341: 405–417. doi:10.1016/j.jmb.2004.06.017

2. Fujii S, Fuchs RP. Defining the position of the switches between replicative and bypass DNA polymerases. EMBO J. 2004;23: 4342–4352. doi:10.1038/sj.emboj.7600438

3. Garibyan L, Huang T, Kim M, Wolff E, Nguyen A, Nguyen T, et al. Use of the rpoB gene to determine the specificity of base substitution mutations on the Escherichia coli chromosome. DNA Repair (Amst). 2003;2: 593–608.
