# Supplementary material for: Processing closely spaced lesions during Nucleotide Excision Repair triggers mutagenesis in E. coli
Source: PLoS Genet. 2017 Jul 7;13(7):e1006881. doi: 10.1371/journal.pgen.1006881 (PMC5521853; doi:10.1371/journal.pgen.1006881)

A. UV-induced mutation spectrum in wild-type strains

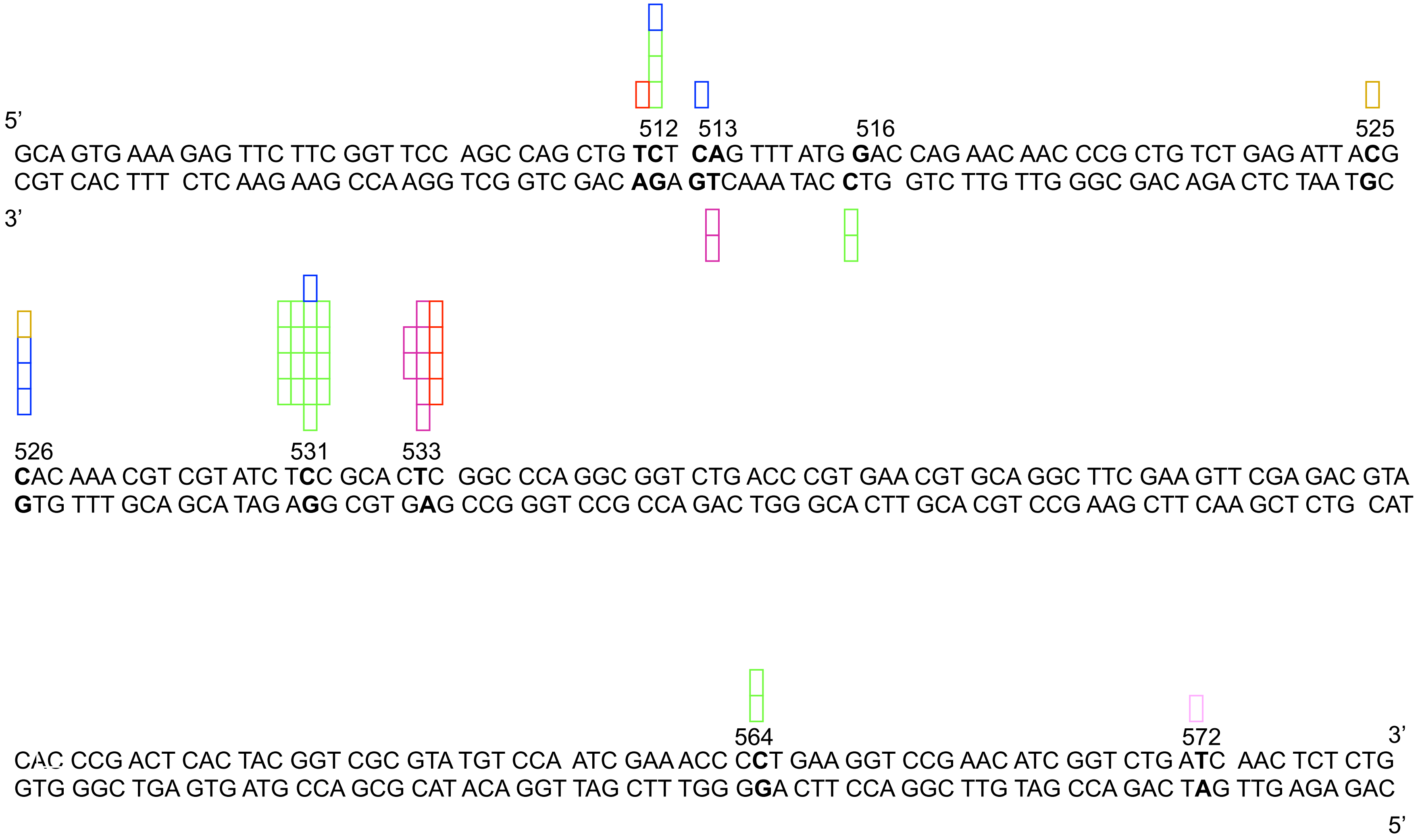

Transition G.C -> A.T A.T -> G.C

Transversion A.T -> T.A G.C -> T.A G.C -> C.G A.T -> C.G

B. UV-induced mutation spectrum in *dinBpolB* strains

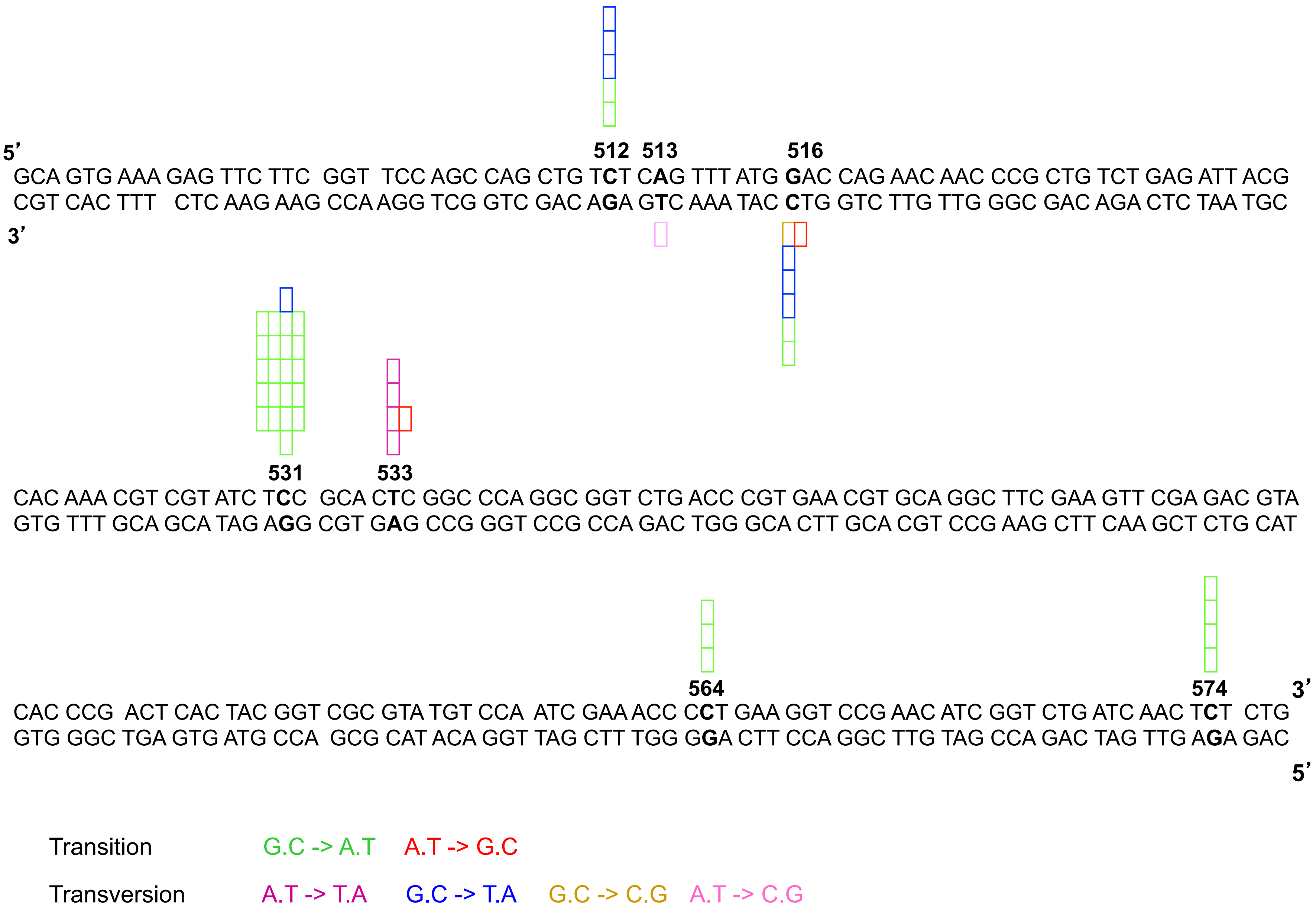

Supplement: S1 Fig — UV-induced mutation spectra in the rpoB gene leading to rifampicin resistance in the wild-type (A) and dinBpolB (B) strains. Each mutant is represented as a rectangle located either above or below the rpoB gene fragment (codons 500–575) depending on the sequence context to match the known preference of UV light to produce lesions at di-pyrimidine sites. The individual base substitutions are color-coded as indicated. (PDF) [file pgen.1006881.s002.pdf]
