# Supplementary figures and images for: Processing closely spaced lesions during Nucleotide Excision Repair triggers mutagenesis in E. coli
Source: PLoS Genet. 2017 Jul 7;13(7):e1006881. doi: 10.1371/journal.pgen.1006881 (PMC5521853; doi:10.1371/journal.pgen.1006881)

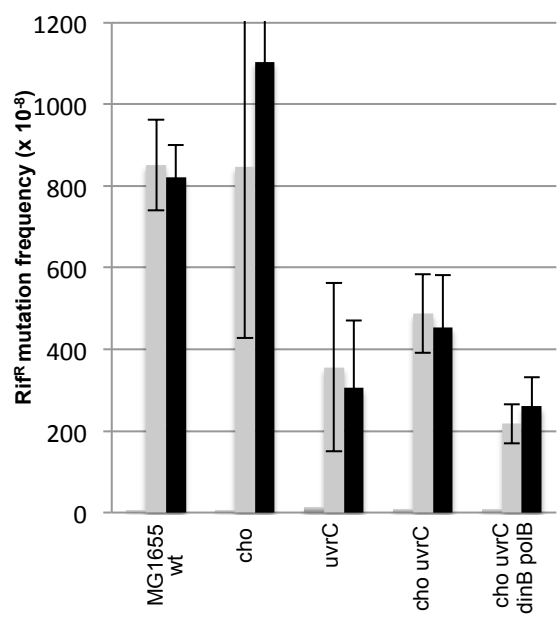

Supplement: S2 Fig — Epistasis of uvrC, but not of its homolog cho to dinBpolB. RifR mutation frequencies were determined in various strains in response to UV irradiation. All strains are constructed in the MG1655 background. To account for the intrinsic differences in UV sensitivity among strains, we compared UV doses leading to similar levels of survival (as in Fig 1A): grey bars correspond to UV doses leading to survival levels ranging between 5–15%, for black bars survival levels range between 1–5% survival. It should be stressed that at these UV doses, the SOS response is fully induced in all strains. White bars represent the level of spontaneous mutation frequency, i.e. no UV irradiation. Average values and standard deviations are plotted for three or more independent experiments per strain. (PDF) [file pgen.1006881.s003.pdf]

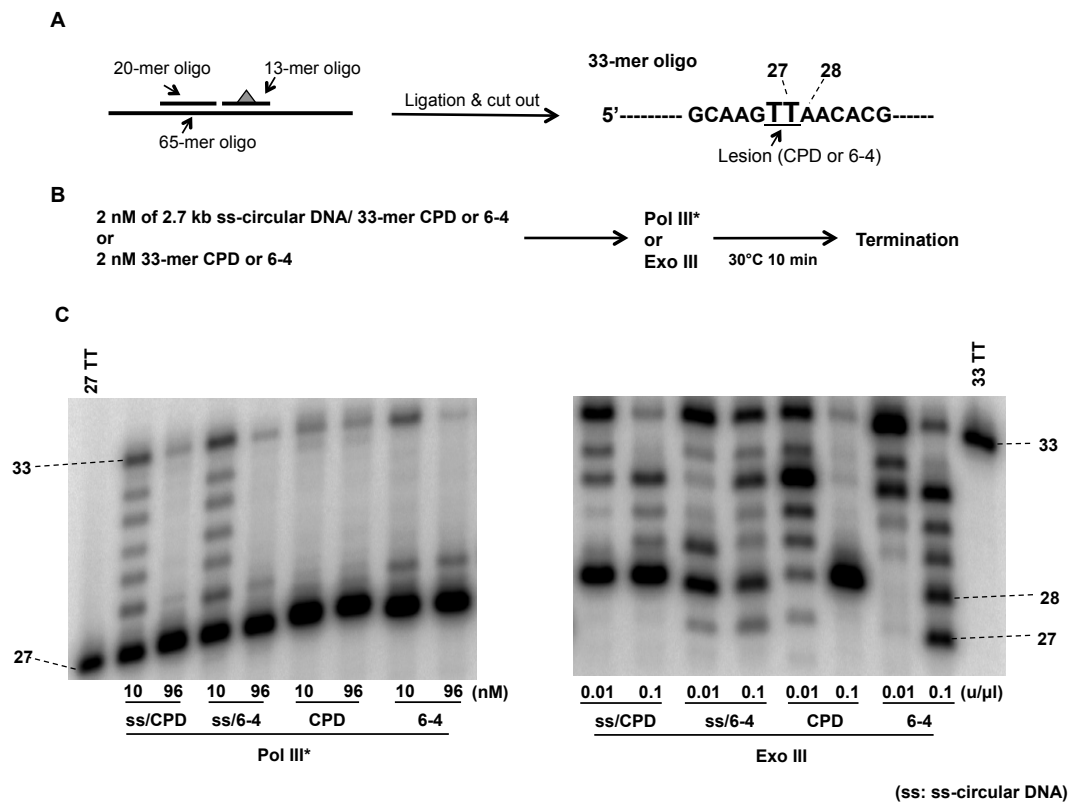

Supplementary Figure 3

Supplement: S3 Fig — In order to get some insight into the specific involvement of Pol IV / Pol II in the gap-filling step during NER-iM, the ability of purified DNA polymerases to elongate primers containing lesions at their 3’-end was investigated. For this purpose, we prepared primers containing lesions at their 3’-end in vitro. A: Preparation of CPD or T(6–4)T containing primers is outlined. The underlined TT in the 33-mer oligo contains either a TT CPD or T(6–4)T. B: The 33-mer oligo or the 33-mer oligo annealed to a 2.7 kb ss-circular DNA are incubated with either Pol III* or Exo III (NEB) for 10 min at 30°C. C: Reaction products from B were analysed by PAGE. Lanes 27 TT and 33 TT are control oligos showing the migration of the 27-mer and 33-mer, respectively. The exonuclease associated with Pol III* clearly generates 27-mer CPD and 6–4 oligos from both substrates. On the other hand, Exo III predominantly generates 28-mer CPD oligo (not 27-mer CPD), while it can convert 28-mer 6–4 oligo into 27-mer. (PDF) [file pgen.1006881.s004.pdf]

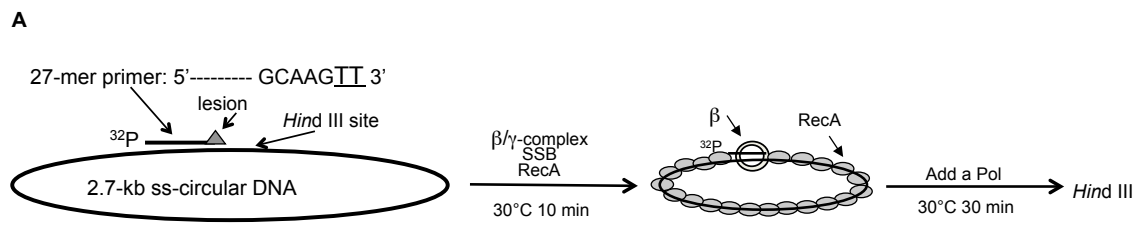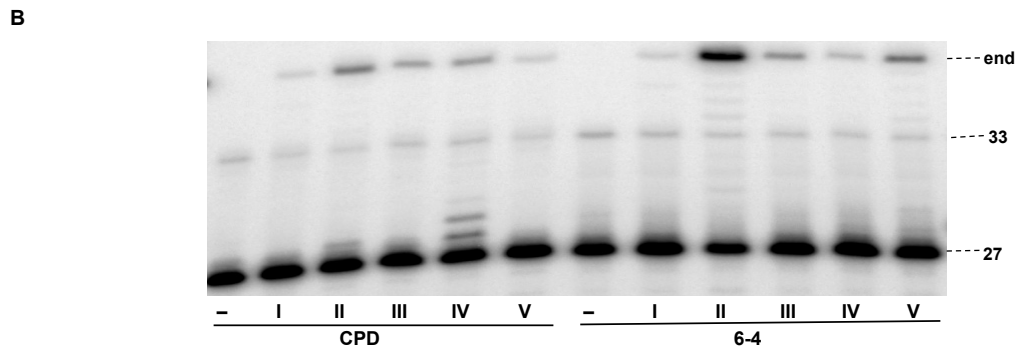

Supplementary Figure 4

Supplement: S4 Fig — Damaged-Primer Elongation (DPE) by E. coli DNA polymerases in vitro: A: Experimental outline for ability of purified DNA polymerases to elongate a primer containing a UV-lesion at its 3’ terminus: The 27-mer CPD or T(6–4)T containing primers (TT CPD or T(6–4)T at the underlined TT sequence), prepared by Pol III* treatment as described in S3 Fig, was annealed on ss-circular DNA. The reaction mixture contains 50 nM β-clamp, 10 nM γ-complex, 10 nM SSB, 2 mM RecA and 2 nM template primer. The 5’ end of the primer is radio-labeled; RecA forms a nucleoprotein filament that is essential for supporting polymerase activity of Pol V [1]. The mixture is incubated for 10 min at 30°C. A given DNA polymerase is added and the mixture is incubated for 30 min at 30°C as indicated. The following amounts of added polymerase correspond to comparable polymerase activity on normal template primer: 5 x 10^−4^ units/μl Pol I KF (USB), 1 nM PolII, 2 nM PolIII*, 4 nM Pol IV or 100 nM Pol V. Finally the reaction product is digested by a restriction endonuclease before PAGE analysis. Detailed procedures have been described previously [1,2]. B: Elongation products analyzed by PAGE following incubation with Pol I to Pol V; “–” represents the control with no addition of polymerase. The abundance of end products generated by the Hind III cleavage relative to Pol I are: for CPD, 1.0 (I), 4.3 (II), 2.5 (III*), 2.8 or 12 (if adding 28/29-mer intermediates) (IV), 1.1 (V); for 6–4, 1.0 (I), > 9.2 (II), 2.8 (III*), 1.4 (IV), 3.8 (V). (PDF) [file pgen.1006881.s005.pdf]

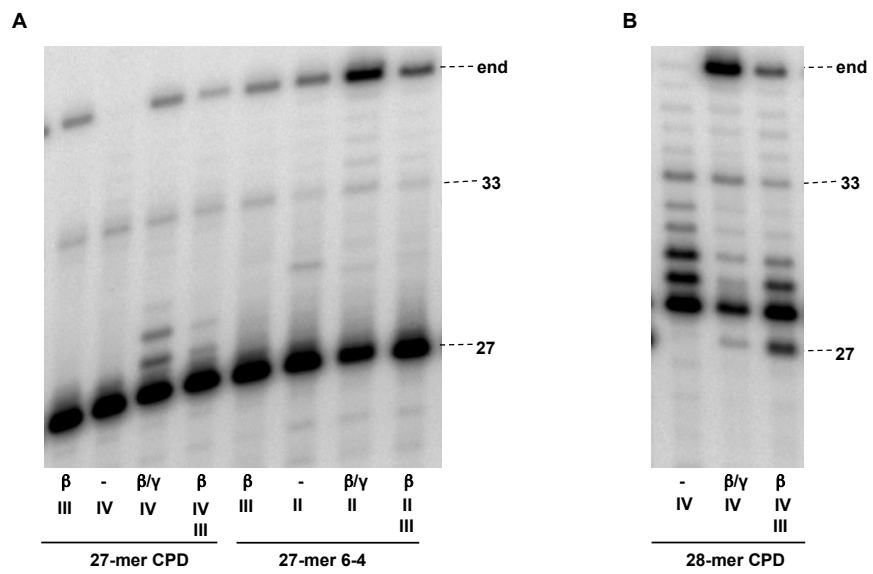

Supplementary Figure 5

Supplement: S5 Fig — The assays were implemented similarly as described in S4 Fig. In addition to 27-mer CPD and 6–4 primers, a 28-mer CPD primer, that was prepared by Exo III treatment as described in S3 Fig, was also tested. From S3 Fig, the best candidates for CPD and 6–4 primer elongations appear to be Pol IV and Pol II, respectively. We focused these Pols for analysing the effect of the β-clamp. A: The presence of the β-clamp is required for efficient 27-mer CPD primer elongation by Pol IV. The β-clamp strongly stimulates elongation by Pol II of the 27-mer T(6–4)T primer. In both cases, while Pol III* mediates β-clamp loading on template DNA, it apparently decreases the efficiency of Pol IV and II elongation product formation. B: In contrast to the elongation of the 27-mer CPD primer, Pol IV is able to elongate the 28-mer CPD primer stepwise even in the absence of the β-clamp. Pol III* again shows negative impact on Pol IV-mediated elongation. (PDF) [file pgen.1006881.s006.pdf]

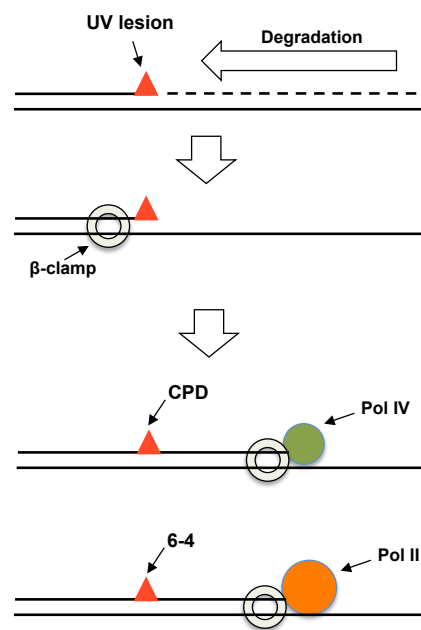

Supplementary Figure 6

Supplement: S6 Fig — Possible roles of Pol II/IV in the context of UV-induced lesions. (PDF) [file pgen.1006881.s007.pdf]
